# Thrombin cleavage of the hepatitis E virus polyprotein at multiple conserved locations is required for genome replication

**DOI:** 10.1371/journal.ppat.1011529

**Published:** 2023-07-21

**Authors:** Danielle M. Pierce, Frazer J. T. Buchanan, Fraser L. Macrae, Jake T. Mills, Abigail Cox, Khadijah M. Abualsaoud, Joseph C. Ward, Robert A. S. Ariëns, Mark Harris, Nicola J. Stonehouse, Morgan R. Herod

**Affiliations:** 1 School of Molecular and Cellular Biology, Faculty of Biological Sciences and Astbury Centre for Structural Molecular Biology, University of Leeds, Leeds, United Kingdom; 2 Discovery and Translational Science Department, Leeds Institute of Cardiovascular and Metabolic Medicine, University of Leeds, Leeds, United Kingdom; 3 Department of Laboratory and Blood Bank, Al Mikhwah General Hospital, Al Mikhwah, Saudi Arabia; University of Maryland, UNITED STATES

## Abstract

The genomes of positive-sense RNA viruses encode polyproteins that are essential for mediating viral replication. These viral polyproteins must undergo proteolysis (also termed polyprotein processing) to generate functional protein units. This proteolysis can be performed by virally-encoded proteases as well as host cellular proteases, and is generally believed to be a key step in regulating viral replication. Hepatitis E virus (HEV) is a leading cause of acute viral hepatitis. The positive-sense RNA genome is translated to generate a polyprotein, termed pORF1, which is necessary and sufficient for viral genome replication. However, the mechanism of polyprotein processing in HEV remains to be determined. In this study, we aimed to understand processing of this polyprotein and its role in viral replication using a combination of *in vitro* translation experiments and HEV sub-genomic replicons. Our data suggest no evidence for a virally-encoded protease or auto-proteolytic activity, as *in vitro* translation predominantly generates unprocessed viral polyprotein precursors. However, seven cleavage sites within the polyprotein (suggested by bioinformatic analysis) are susceptible to the host cellular protease, thrombin. Using two sub-genomic replicon systems, we demonstrate that mutagenesis of these sites prevents replication, as does pharmacological inhibition of serine proteases including thrombin. Overall, our data supports a model where HEV uses host proteases to support replication and could have evolved to be independent of a virally-encoded protease for polyprotein processing.

## Author summary

Positive-strand RNA viruses produce polyproteins that are cleaved by proteases that control viral replication. The polyproteins of all well studied positive-strand viruses undergo proteolysis in a highly controlled manner to generate functional proteins and regulate the transition from translation to RNA replication. Proteolysis of viral polyproteins is generally performed by virally-encoded proteases, although host cellular proteases are used by some viruses. In this report, we provide evidence that suggests that hepatitis E virus, a medically important human pathogen, does not encode a protease and unlike other viral polyproteins, cannot undergo auto-catalytic processing. Instead, we provide evidence that the polyprotein is susceptible to proteolysis by the host cell protease thrombin and that this is essential for viral replication. Our data contradict the previous dogma of positive-sense viral replication and suggests a model where this virus could have evolved to use a host protease to control viral replication and tropism.

## Introduction

Hepatitis E virus (HEV) is a leading cause of acute viral hepatitis [1,2]. There are an estimated 20 million cases of HEV each year that contribute to >3% of all virus related hepatitis mortalities [3, 4]. Human HEV is part of the *Hepeviridae* family of viruses, within the *Paslahepevirus* genus. The genus has two currently recognised species, *P*. *alci* and *P*. *balayani*, the latter of which has been identified in a wide range of animals and humans and is currently sub-classified into eight genotypes (G1 –G8) [5–7]. G1 and G2 viruses appear to be obligate human pathogens that are transmitted between humans faecal-orally, with the potential to cause large outbreaks. Viruses in G3 and G4 have been isolated in several animal species and are believed to be zoonotically transmitted to humans [8–10]. These viruses are of particular concern and have been suggested to exist within a reservoir of domestic and wild porcine species where they can be transmitted to humans, for example, via poorly prepared pork products [11]. HEV infection is usually self-limiting, however, infection during pregnancy, especially with G1 viruses can give rise to significant mortality of up to 30% [12]. A HEV vaccine is currently only approved in China, with other treatment options such as ribavirin and PEG-α-interferon available in other parts of the world [13]. HEV is therefore not only a significant global healthcare problem but also imposes risks to the farming industry and food chain security.

HEV is a positive-sense single-stranded RNA virus. The genome contains three open reading frames (ORF) [6]. ORF1 is translated into the viral polyprotein (pORF1) that is necessary and sufficient for viral RNA replication. The second and third open reading frames, ORF2 and ORF3, are translated into the viral capsid protein and a small membrane protein involved in virus release, respectively. An additional fourth open reading frame, ORF4, has also been identified in G1 viruses [14]. Alphavirus replication of the viral genome is mediated by the pORF1 polyprotein. Through sequence homology to related virus families, such as the caliciviruses and togaviruses, pORF1 was predicted to contain at least six distinct protein domains [15]. Recent functional and structural studies have suggested refinements to these initial predictions. At the N-terminus of the polyprotein is a methyltransferase (MeT) domain demonstrated to have methyltransferase activity and to interact with the adjacent Y domain [16–18]. Other studies have suggested a portion of the Y domain forms part of the MeT “iceberg region” with structural prediction suggesting together they could form a capping pore equivalent to alphavirus NSP1 [16,18]. At the C-terminus of the Met-Y domains is the putative cysteine protease (PCP) originally suggested to be a protease responsible for pORF1 proteolysis discussed further below. In the centre of the polyprotein is a region of high sequence diversity, termed the hyper-variable region (HVR) with poor sequence homology and no known function, with some studies suggesting this can act as a hinge to allow pORF1 flexibility and also influence viral replication efficacy [19–21]. The HVR is followed by a macrodomain or X region that can bind ADP-ribose and has been suggested to be equivalent to NSP3 from alphaviruses but no biochemical activity has thus far been determined [18,22,23]. At the C-terminus of the polyprotein are domains with helicase (Hel) and RNA-dependent RNA-polymerase (RdRp) activity [24–30].

The polyproteins of well-studied positive-sense RNA viruses, such as the flaviviruses, caliciviruses and alphaviruses have been shown to undergo processing to generate the functional protein subunits (sometimes called replicase or non-structural proteins) [31–34]. Several studies have attempted to understand if and how HEV pORF1 undergoes processing but with varying results. Studies using heterogenous expression systems have suggested auto-catalytic processing of pORF1, potentially mediated by the viral PCP region, to generate smaller protein fragments, but with inconsistent results [35–41]. These studies also implicate one or more cysteine residues in the PCP as important for this proteolysis. Furthermore, a recent study reported pORF1 processing when expressed either in a non-replicative form or from a sub-genomic replicon and infectious molecular clone, however, the protease responsible was not identified [42]. The data reporting pORF processing are confused by the recent structure of the PCP region derived by X-ray crystallography, which revealed a resemblance to a fatty acid binding protein [43]. Further data suggesting the PCP region can chelate zinc, has deubiquitinase activity, and acts with the upstream Met-Y-domain also question the function of this domain [43–45]. Other studies have suggested that pORF1 cannot be processed in heterogenous systems and is not processed in transfected cells, and therefore, the intact precursor is hypothesised to be functionally active [46].

In addition to auto-catalytic pORF1 processing, one investigation suggested that the host cellular protease thrombin could play a role in processing pORF1 and implicated processing at two locations within pORF1 [47]. Thrombin is synthesised in hepatocytes as the zymogen prothrombin and secreted into the blood system [48]. In blood coagulation during haemostasis (the stemming of bleeding), factor Xa and factor Va combine on phosphatidylserine containing membranes to form prothrombinase which cleaves prothrombin at two sites to yield thrombin and prothrombin fragments 1+2 [48,49]. The use of host proteases to control virus processing and replication is not unique, with many positive-sense viral polyproteins cleaved in places by cellular proteases in conjunction with virally-encoded proteases. For example, noroviruses use caspases to control processing of the NS1/2 non-structural protein to generate individual proteins NS1 and NS2 [50,51] and hepatitis C virus uses signal peptide peptidase and signal peptidase for polyprotein processing [52].

The goal of this study was to understand the processing of the HEV pORF1 and its importance for viral replication. Our data suggests that, unlike many other RNA viruses, the HEV pORF1 does not have any intrinsic auto-proteolytic activity and predominantly generates a ~190 kDa precursor. However, sequence alignments identified seven conserved potential thrombin cleavage sites within pORF1, six of which we demonstrated were susceptible to thrombin proteolysis. Furthermore, we demonstrate that mutagenesis of these cleavage sites and pharmacological inhibition of thrombin are able to prevent viral replication. Thus, our data support a model where hepatocyte-specific host proteases are essential for HEV replication.

## Results

### The HEV pORF1 polyprotein contains multiple potential thrombin cleavage sites

**Fig 1:**
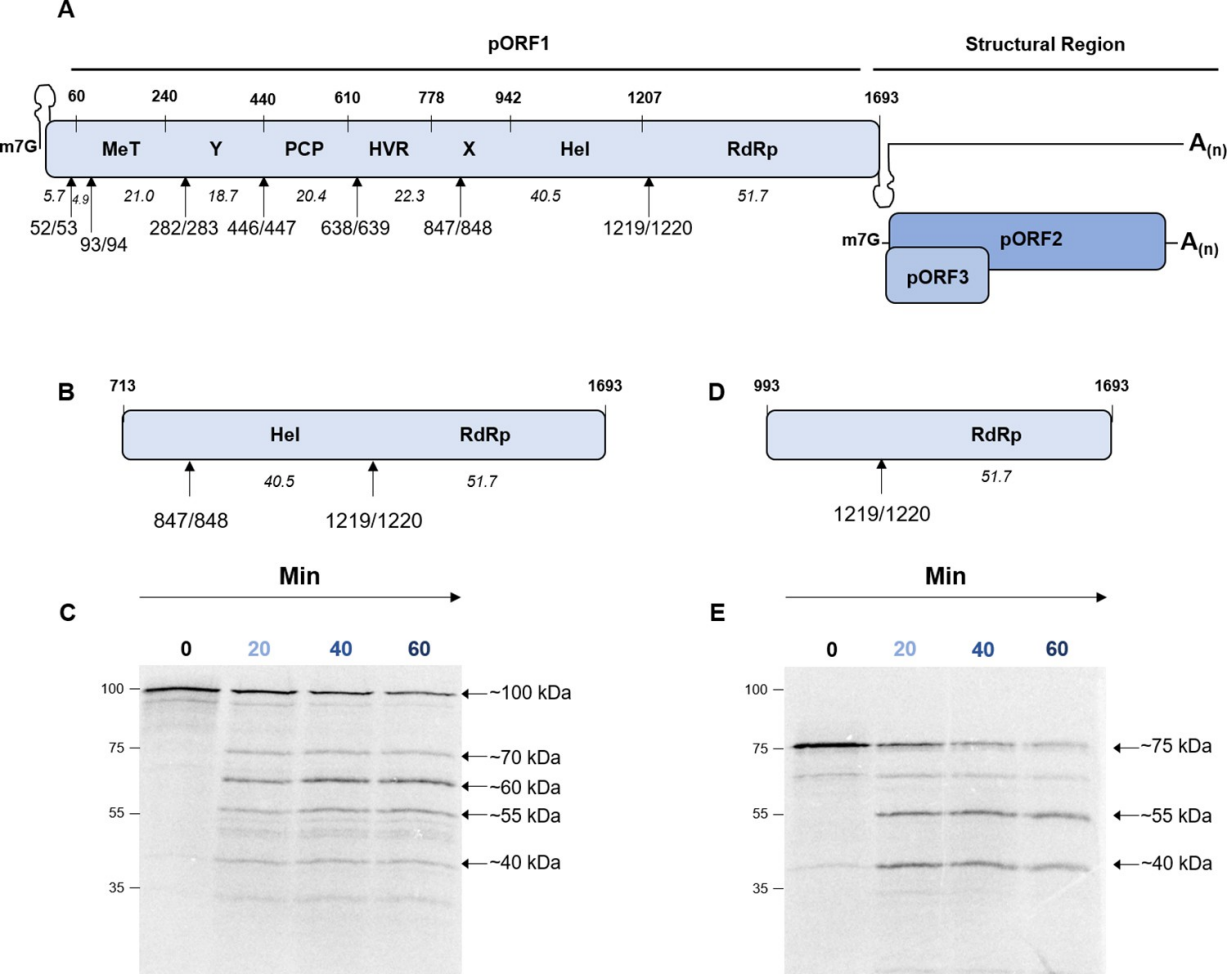
Thrombin proteolysis of the C-terminal portion of pORF1. **(A)** Schematic of the HEV genome showing the three ORF, with the pORF1 polyprotein labelled with the position of the seven predicated functional domains. The location of the conserved thrombin recognition sequences in pORF1 is indicated. The numbers in italics indicate the predicted molecular weight of products after cleavage at these positions. All numbers are the amino acid positions in the Sar55 sequence (GenBank reference AF444002). **(B)** and **(D)** Schematics of the truncated pORF1 polyprotein expression construct in used in **(C)** and **(E).** Plasmids expressing C-terminal portions of the pORF1 polyprotein were used to template *in vitro* coupled transcription/translation reactions labelled with [^35^S] methionine. A zero minute sample was taken before the addition of 0.5 IU of thrombin followed by collection of protein samples at the indicated time-points representing minutes after the addition of thrombin. Samples were harvested into Laemmli buffer to stop reactions, proteins separated by SDS-PAGE and visualised by autoradiography and phosphorimaging. The approximate molecular weight of each product is indicated together with the molecular weight ladder in kDa on the left of each gel.

There is conflicting evidence in the literature as to if and how the HEV pORF1 is proteolytically processed to functional proteins. Using a replicon system, Kanade *et al* demonstrated that mutation of two thrombin cleavage junctions in pORF1 prevented replication, consistent with a role for thrombin in processing of the polyprotein [47]. Both of these cleavage sites (Fig 1A) have the proline-arginine residue pair that constitutes a typical thrombin cleavage site and fits within the broader thrombin recognition sequence [53]. We extended this analysis by aligning the ~2000 genotype 1–4 HEV sequences currently available and identified an additional five potential thrombin cleavage junctions (Fig 1A) that contain a core recognition sequence and would be compatible with the extended thrombin recognition sequence. Furthermore, of these five additional sites, four are highly conserved across *Paslahepevirus* sequences (S1 Fig).

### Thrombin proteolysis of a pORF1 C-terminal portion

Of the seven potential thrombin sites identified in our analysis, two (at PR847/848 and PR1219/1220, where the numbers refer to the amino acid position within pORF1 of the P and R residues that are essential for thrombin proteolysis) have already been shown to be susceptible to proteolysis *in vitro*, using purified enzyme and a shortened cleavage junction substrate [47]. First, it was important to establish that we could detect processing of these previously described substrates by thrombin in our assays. In contrast to the previous study, we chose to express these cleavage sites as larger portions of pORF1. We reasoned that expression of the cleavage junctions as part of a larger polyprotein portion would present them in a more native environment.

To this end, we generated two T7 expression constructs expressing two C-terminal portions of the genotype 1 HEV pORF1 (Sar55 isolate) polyprotein. The first portion expressed amino acids 713–1693 and contained both cleavage junctions at PR847/848 and PR1219/1220, which have been previously suggested to be thrombin substrates. The second portion expressed amino acids 993–1693 and thus only contained the cleavage junction at PR1219/1220. Both of these constructs were used for *in vitro* coupled transcription and translation pulse chase experiments to allow the detection of both final products and any processing intermediates. To a duplicate set of reactions, purified human thrombin was added 20 minutes after the start of the chase. Protein samples were taken at regular intervals, separated by SDS-PAGE, and analysed by autoradiography (Figs 1B–1E and S2).

In the absence of thrombin, both truncated precursors predominately generated one full-length precursor (of ~100 kDa or ~75 kDa for the 713–1693 or 993–1693 portions, respectively), consistent with the idea that this portion of the polyprotein contains no protease activity. Upon the addition of thrombin, the 713–1693 portion was processed into four products of ~70, ~60, ~55 and ~40 kDa. The products at ~60, ~55 and ~40 kDa increased in abundance and would correspond well to the predicted molecular weights after cleavage at either PR1219/1220 alone or at both PR847/848 and PR1219/1220. The unidentified product at ~70 kDa was less abundant and decreased in intensity over the time course. Upon the addition of thrombin, the 993–1693 portion was processed to generate a product at ~ 55 kDa. It therefore seems likely that for processing of both the 713–1693 and 993–1693 portions the ~55 kDa product is the result of processing at the PR1219/1220 predicted thrombin cleavage junction. The 993–1693 portion also generated a product that we could not identify at ~40 kDa.

### Thrombin proteolysis of the N-terminal pORF1 portion

**Fig 2:**
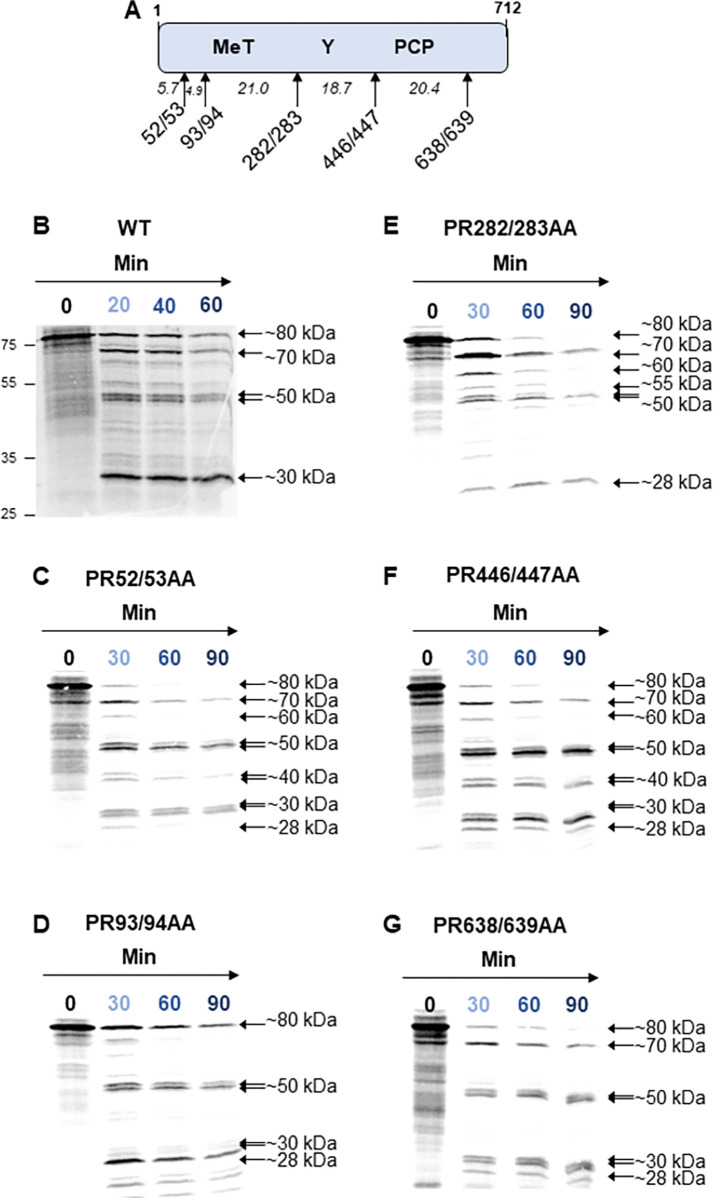
Thrombin proteolysis of the N-terminal portion of pORF1. **(A)** Schematic of the truncated pORF1 expression plasmid. **(B)** A plasmid expressing the N-terminal portion of the WT pORF1 polyprotein were used to template *in vitro* coupled transcription/translation reactions labelled with [^35^S] methionine. A zero minute sample was taken before the addition of 0.5 IU of thrombin followed by collection of protein samples at the indicated time-points representing minutes after the addition of thrombin. **(C-G)** Plasmid expressing amino acids 1–712 of pORF1 with the indicated alanine substitutions at amino acids **(C)** PR52/53, **(D)** PR93/94, **(E)** PR282/283, **(F)** PR446/447, **(G)** PR638/639, before being used to template [^35^S] methionine labelled *in vitro* coupled transcription/translation. A zero minute sample was taken before the addition of 0.5 IU of thrombin followed by collection of protein samples at the indicated time-points representing minutes after the addition of thrombin. Samples were harvested into Laemmli buffer to stop reactions, proteins separated by SDS-PAGE and visualised by autoradiography and phosphorimaging. The approximate molecular weight of each product is indicated together with the molecular weight ladder on the left of the gel.

After analysing processing of the C-terminal portion of pORF1, we turned our attention to the N-terminal portion. This portion contains five predicted thrombin cleavage junctions, therefore processing is likely to be more complex. A T7 expression construct was generated expressing amino acids 1–712 which contained all five predicted thrombin cleavage junctions. We analysed this N-terminal region of the polyprotein using *in vitro* coupled transcription and translation in the presence or absence of thrombin (Figs 2 and S3).

Firstly, before the addition of thrombin this polyprotein was visualised as a single protein that is approximately the predicted molecular weight of the uncleaved product. Therefore, in common with the data above for the C-terminal region, the N-terminal portion of the polyprotein was also unable to undergo significant auto-catalytic proteolysis, despite including the putative viral protease (PCP).

Upon the addition of thrombin, the protein underwent proteolysis to produce at least five new products. Of these new products the largest, of ~70 kDa, seems likely to result from cleavage of ~10 kDa from the N-terminus of the polyprotein, which is consistent with processing at the predicted PR93/94 site. Cleavage of the construct generated products at ~30 kDa and ~55 kDa, suggesting these products have N- and C-termini within the first 712 amino acids of pORF1. The generation of these products suggest at least some, if not all, of the predicted sites at amino acids PR52/53, PR282/283, PR446/447 and PR638/639 are susceptible to thrombin proteolysis. Using the predicted molecular weight of the different cleavage products, it seemed likely that the ~30 kDa product was the result of processing at PR282/283, and the ~55kDa product was the result of processing at PR446/447. However, these products could have arisen through multiple combinations of proteolysis events. For example, the ~30 kDa product could be the result of proteolysis at both the PR93/94 and PR446/447 sites simultaneously. It was therefore still difficult to establish whether all of the predicted sites were cleaved by thrombin from the data generated in these assays alone.

To help elucidate these possible cleavages further, we used site directed mutagenesis to introduce alanine substitutions at either the PR52/53, PR93/94, PR282/283, PR446/447 or PR638/639 residue pairs within the context of the 1–712 precursor. These substitutions were designed to prevent recognition and proteolysis by thrombin. This generated a new series of T7 expression constructs in which each putative protease site was removed. This new series of constructs was used for *in vitro* transcription and translation in the presence or absence of thrombin as above (Fig 2C–2G and S3). To understand the effect of each mutation on processing, the relative proportion of each product was quantified and compared to the wild-type (WT) control. For ease of interpretation, the main differences between the different constructs were plotted in comparison to WT (S4 Fig). Importantly, if we removed a genuine thrombin cleavage site, we would anticipate that the products formed by proteolysis would be different to the WT control (i.e., a disappearance of smaller products and concomitant accumulation of larger protein products). However, if the site was not genuinely susceptible to thrombin mediated proteolysis, then we would expect to see no difference in the pattern of proteolysis compared to the WT control.

Mutation of the PR93/94 position decreased the rate of processing of the ~80 kDa (i.e., full-length) protein, and slowed the appearance of products at ~30 kDa and ~70 kDa. These observations therefore suggest that processing at the PR93/94 position could take place and is key for generating the ~70 kDa product.

Mutation at the PR282/283 position slowed the appearance of the ~30 kDa product, as well as generating a novel product at ~55 kDa, not observed with any of the other constructs. These data suggest processing at the PR282/283 site could occur and was important for generating the ~30 kDa product.

The PR446/447AA mutation increased the appearance of the ~40 kDa product, and moderately changed the appearance of the ~30 kDa product. Thus, these data suggest that processing could occur at this position, with the ~40 kDa product the result of cleavage at PR282/283 and possibly at PR638/639.

Similar changes were observed with either the PR52/53AA or PR638/639AA mutations in comparison to the WT but these were less pronounced. This could either be due to a lack of efficient cleavage at these positions, or the resultant differences being too small to visualise in these assays. Taken together, these data provide evidence for processing at five predicted thrombin cleavage sites, PR93/94, PR282/283, PR446/447, PR847/848 and PR1219/1220.

### *In vitro* proteolysis of pORF1

Our experiments could not detect auto-catalytic activity of pORF1 portions in an *in vitro* transcription and translation system, in contrast to many other viruses [54,55]. To verify that no auto-catalytic processing was also observed when full-length pORF1 was expressed, a T7 expression construct was generated expressing the entire pORF1 coding sequence (using the same G1 Sar55 isolate). As a control to confirm that auto-catalytic processing is possible in this system we generated an equivalent construct expressing the non-structural ORF1 polyprotein from murine norovirus (MNV). This viral polyprotein was chosen based on its known processing profile, similar layout of functional domains and comparable polyprotein length [50,56,57]. These two expression constructs were used to template *in vitro* coupled transcription and translation, labelled with [^35^S] methionine. As before, samples were taken at regular time points, proteins separated by SDS-PAGE and analysed by autoradiography (S3 and S5 Figs).

**Fig 3:**
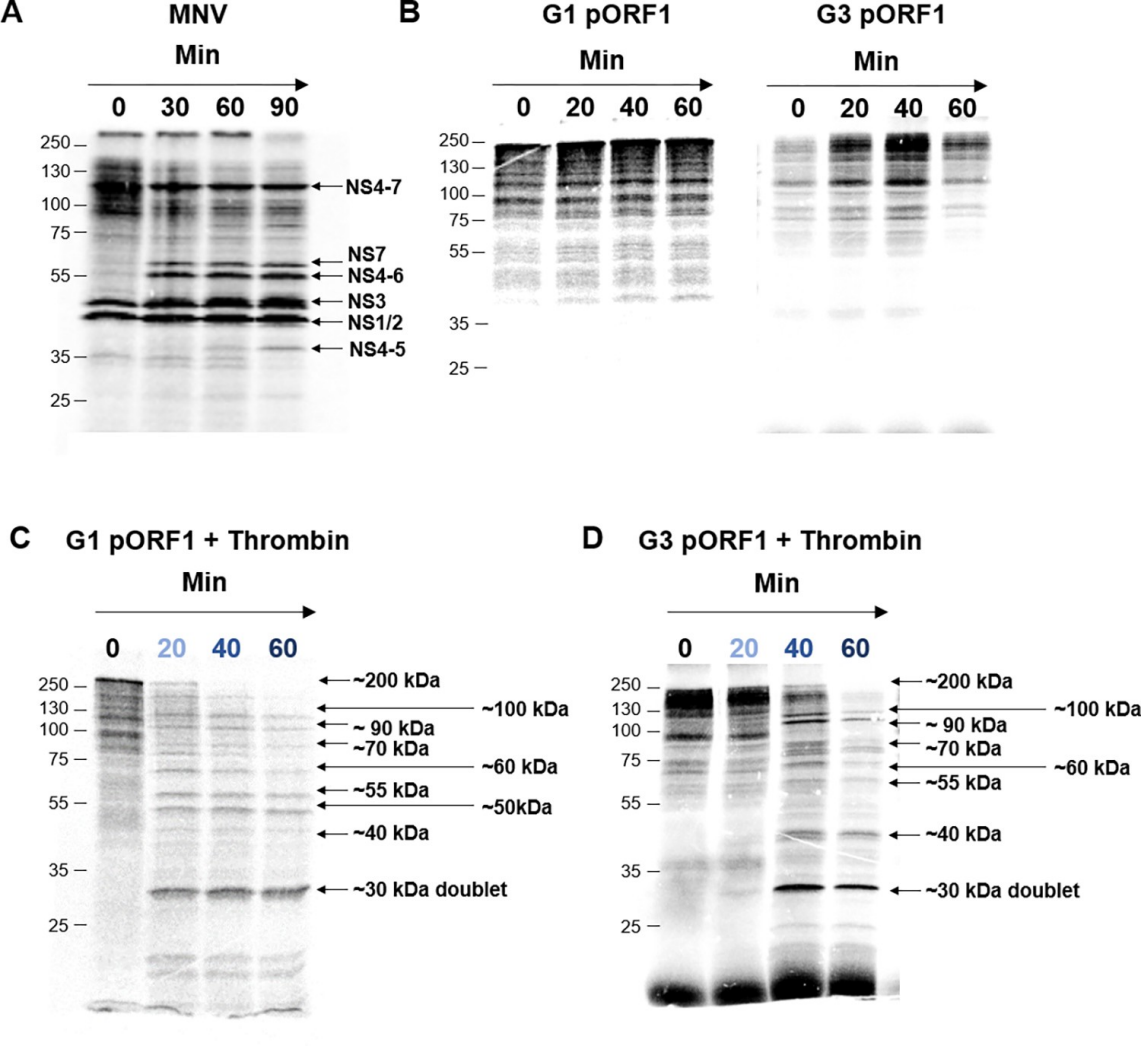
Thrombin proteolysis of pORF1. Plasmids expressing MNV polyprotein **(A)** or the HEV G1 and G3 pORF1 **(B)** were used to template *in vitro* coupled transcription/translation reactions labelled with [^35^S] methionine. Samples were taken at regular intervals, reactions stopped by the addition of Laemmli buffer, proteins separated by SDS-PAGE and visualised by autoradiography and phosphorimaging. The identity of MNV products is indicated together with the molecular weight ladder on the left of each gel. To a reaction with HEV G1 pORF1 **(C)** or G3 pORF1 **(D)** a zero minute sample was taken before the addition of 0.5 IU of thrombin followed by collection of protein samples at the indicated time-points representing minutes after the addition of thrombin. Samples were harvested into Laemmli buffer to stop reactions, proteins separated by SDS-PAGE and visualised by autoradiography and phosphorimaging. The product of thrombin proteolysis indicated together with their approximate molecular weight are indicated.

With the MNV control plasmid, at least six distinct protein products could be observed which correlated well to a range of different mature and precursor protein products that would occur from auto-catalytic proteolysis (Fig 3A) [50,56–58]. These data demonstrate that auto-catalytic processing is possible in this *in vitro* system.

In contrast, translation of pORF1 produced predominantly just one product of approximately the predicted size for full-length unprocessed HEV pORF1 (Fig 3B). There was a smaller abundance of lower molecular weight products produced that we attribute to early termination events, which is common when performing *in vitro* translation of large proteins [59]. To determine if processing occurred but was very slow compared to MNV we extended the incubation period to 24 hours, however, there was still no change in the pORF1 products over this extended time course. This result was consistent with the hypothesis that full-length pORF1 has no intrinsic auto-catalytic activity. In an attempt to stimulate any intrinsic protease activity of pORF1, we added divalent metal ions or fatty acids, both of which have been suggested to interact with the PCP domain however, none of these approaches stimulated proteolysis in this assay [43]. Finally, we investigated the products formed from thrombin mediated proteolysis of full-length pORF1 (Fig 3C). Upon the addition of exogenous thrombin, pORF1 underwent proteolysis to generate at least nine distinct additional protein products, each of which were quantified (S5B Fig). There was also a clear decrease in the full-length pORF1 upon the addition of thrombin. Larger molecular weight products, at ~100, ~90 and ~70 kDa decreased in relative abundance over the duration of the experiment. The relative abundance of products at ~60 kDa and ~40 kDa did not significantly change. There was a small increase in the abundance of the product at ~55 kDa, although this was not significant over the duration of the experiment, and a clear increase in the abundance of the smaller protein products at ~50 kDa and the doublet at ~30 kDa.

To determine if thrombin was able to process the pORF1 from other genotypes we next generated a T7 expression construct expressing the entire pORF1 coding sequence from the genotype 3 G3-HEV83-2-27 isolate, which was used *in vitro* coupled transcription and translation, labelled with [^35^S] methionine and imaged as before (Fig 3D). As with the G1 construct upon the addition of exogenous thrombin, G3 pORF1 underwent similar proteolysis to generate at multiple distinct additional protein products. Larger molecular weight products, at ~100 and ~90 kDa decreased in relative abundance over the duration of the experiment. As for G1 pORF1, there was a clear increase in the abundance of the smaller protein products, in particular at ~40 kDa and the doublet at ~30 kDa. These data suggested thrombin is able to process pORF1 from different HEV genotypes to generate a similar array of products.

### Immunoprecipitation of polyprotein products

**Fig 4:**
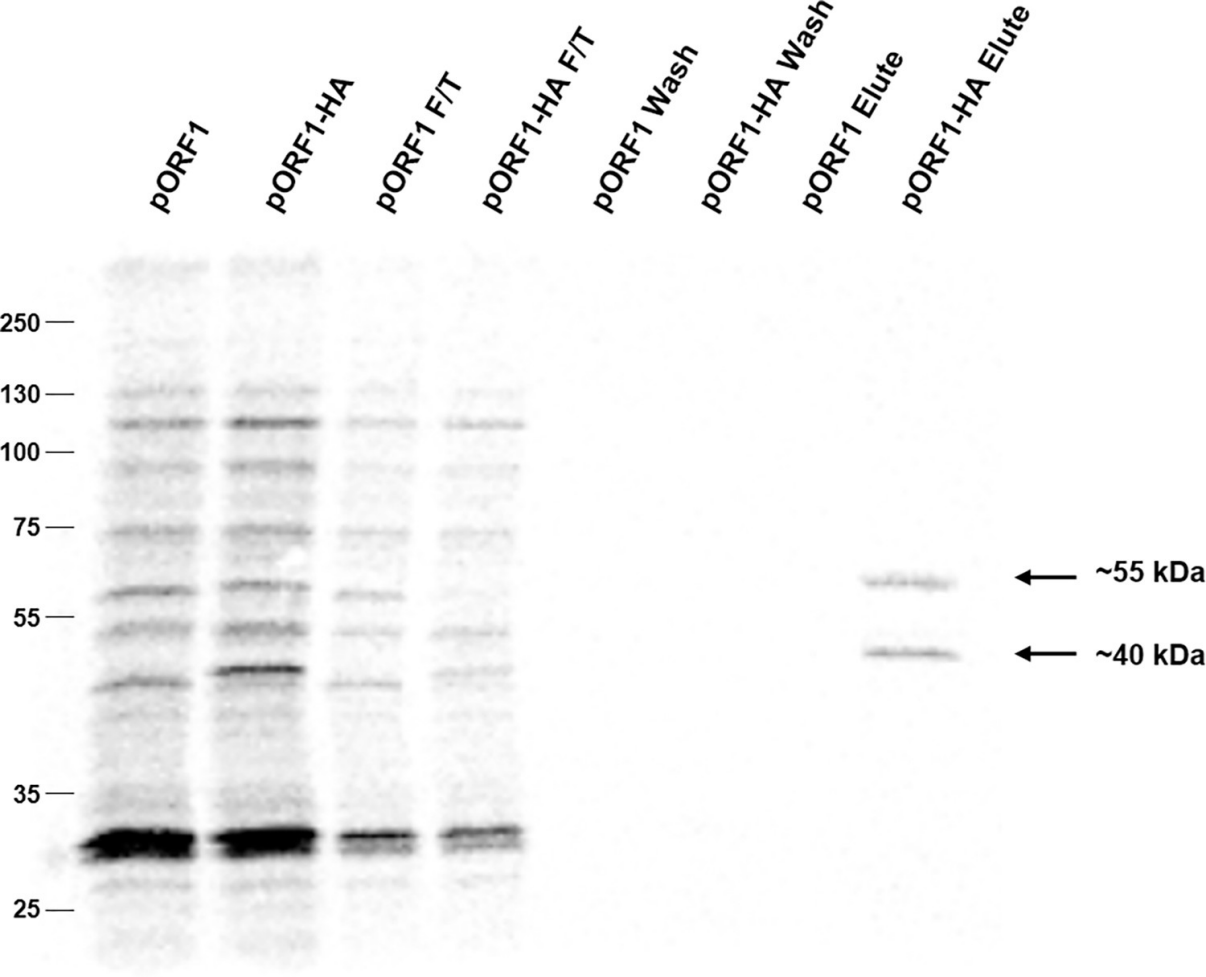
Immunoprecipitation of HEV pORF1 products. Plasmids expressing HEV pORF1 or pORF1-HA containing a C-terminal HA-tag were used to template [^35^S] Met labelled pulse-chase *in vitro* coupled transcription/translation reactions. Reactions were incubated with thrombin for 90 minutes before proteins were immunoprecipitated with anti-HA antibody. The pre-IP samples, flow through (F/T), wash and elute samples were separated by SDS-PAGE and visualised by autoradiography. The immunoprecipitated ~40 kDa and ~55 kDa products are indicated together with the molecular weight ladder on the left of the gel. Representative result from one of three independent experiments.

A notable observation from our *in vitro* processing data was that there were more than the seven cleavage products that would be predicted from full proteolysis. We hypothesised therefore that some of these additional products were protein intermediates. Therefore, we sought to identify the composition of some of these potential precursors in addition to fully processed proteins. Due to the lack of suitable antibody reagents for immunoprecipitation, we adapted the G1 pORF1 T7 expression vector by incorporating a HA-tag at the C-terminus of pORF1 to allow immunoprecipitation of RdRp containing products and precursors. This expression vector was used in an *in vitro* coupled transcription and translation assay with [^35^S] methionine before HA-containing products were immunoprecipitated, separated by SDS-PAGE and imaged by autoradiography (Fig 4).

Thrombin-mediated processing of HA-labelled pORF1 yielded at least nine additional products, which are approximately equivalent to the untagged pORF1 plasmid and similar to the products observed in earlier pulse-chase experiments. Notably, some products in the pORF1-HA sample appeared to have a marginally greater molecular weight compared to the unlabelled pORF1 sample, as may be anticipated with a C-terminal epitope extension. Immunoprecipitation of the HA-tagged sample yielded two clear products of ~40 kDa and ~55 kDa. It seems likely that the ~55 kDa product corresponds to the C-terminal fragment generated from cleavage at the PR1219/1220 position, thus corresponding to the RdRp domain. The smaller ~40 kDa product would be consistent with the results in Fig 1 and could be the result of inadvertent off-target proteolysis, incorrect translation initiation or a co-immunoprecipitated product.

### Preventing thrombin proteolysis inhibits viral replication

**Fig 5:**
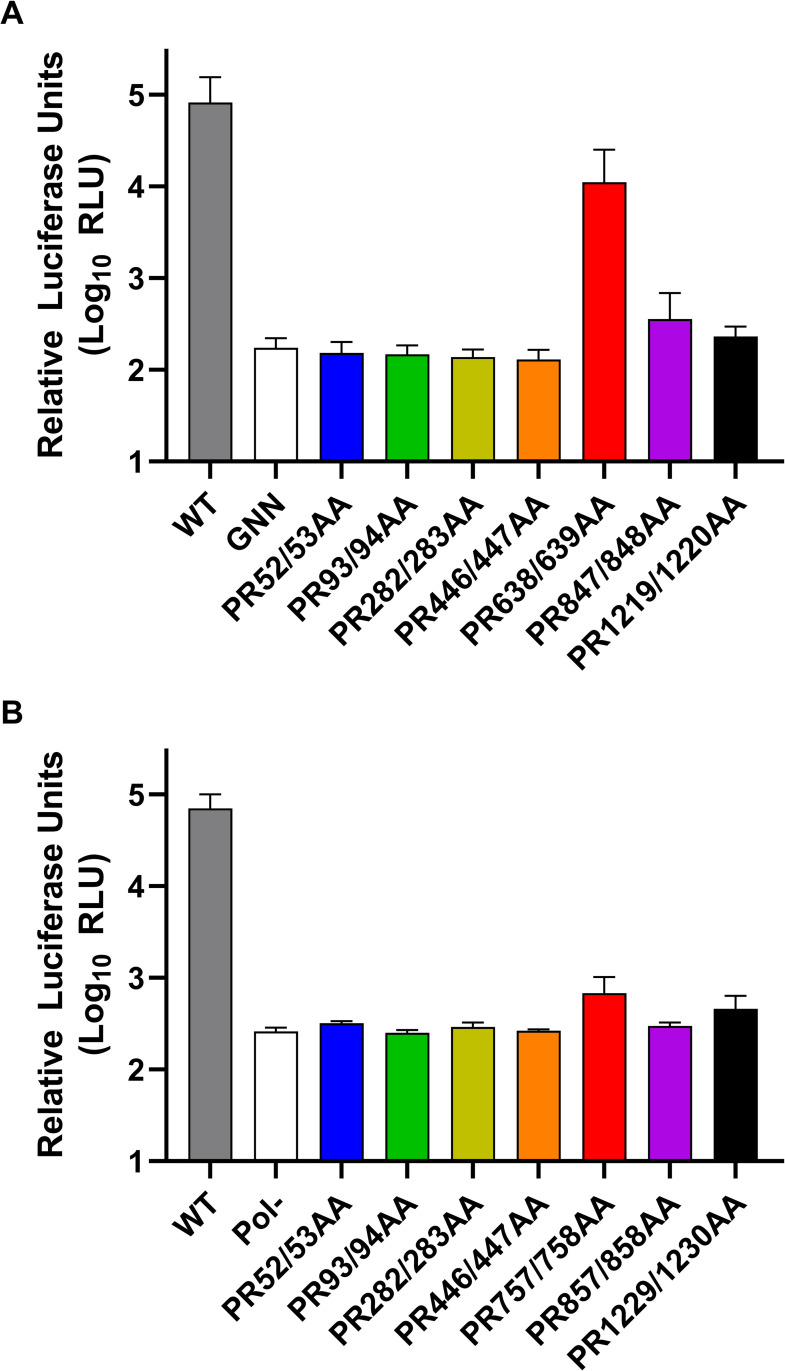
Preventing thrombin proteolysis abrogates HEV replication. Huh7 cells were electroporated with **(A)** G1 or **(B)** G3 HEV replicon RNA containing the indicated mutations at predicted thrombin cleavage junctions, in addition to the WT and GNN/Pol- control replicons. Cells were harvested at 96 h post-electroporation and luciferase activity determined. Data shown represents log_10_ of mean relative luciferase activity at 96 h post-electroporation (n = 3 +/- SEM).

To understand if any of these sites are important for virus replication, we utilised sub-genomic replicons (SGR) of both the G1 Sar55 HEV isolate and G3 HEV83-2-27 isolate, both of which contained a nano-luciferase (nLuc) reporter sequence in place of the viral structural proteins. Monitoring the production of luciferase allows for indirect quantification of virus replication. The SGRs were modified by alanine substitution of each proline arginine pair, to prevent recognition and proteolysis by thrombin. This generated seven new constructs for both G1 and G3 replicons each with an individual proline arginine amino acid pair mutated (termed PR52/53AA, etc). RNA from these replicons were electroporated into Huh7 cells along with a wild-type (WT) control replicon or a replicon containing an inactivating mutation to the RdRp (for G1 this was an active site mutation, termed GNN, while for G3 this was a frame shift mutation, termed Pol-), and luciferase activity was monitored over four days to measure RNA replication (Figs 5 and S6).

Both WT HEV replicons demonstrated a >100-fold increase in luciferase activity over the duration of the experiment compared to the replication defective (GNN or Pol-) controls. For the G1 replicon, electroporation of all but one of the seven replicons with proline-arginine substitution significantly impaired replication, with luciferase activity equivalent to the replication-defective control (GNN). The exception was PR638/639AA which demonstrated an approximate 2-fold reduction in luciferase activity compared to the WT replicon, although this was not statistically significant. For the G3 replicon, all of the seven proline-arginine substitutions significantly prevented replication, with luciferase activity equivalent to the replication-defective control (Pol-). These data would suggest that all but one of the PR residues where thrombin is predicted to cleave are essential for both G1 and G3 replication.

### Inhibition of serine proteases prevents HEV replication

**Fig 6:**
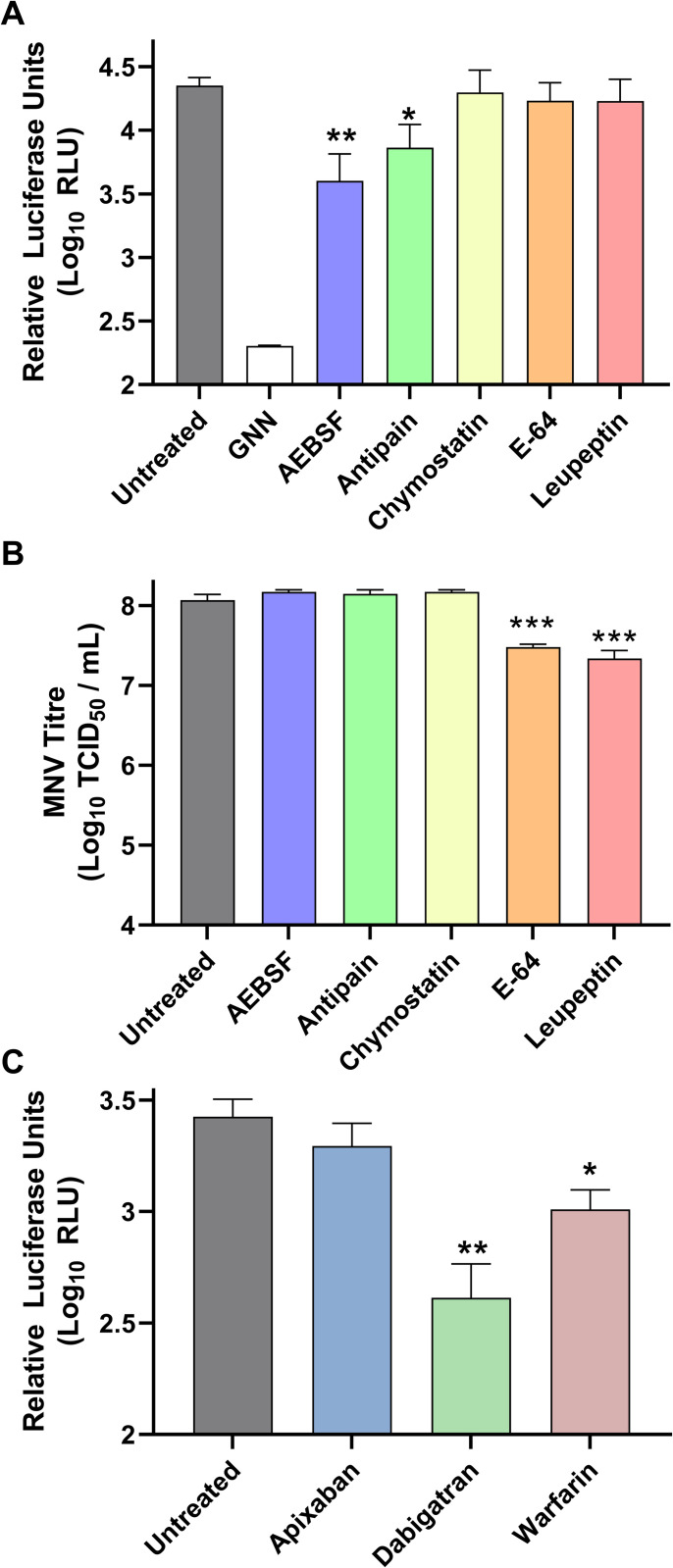
Pharmacological inhibition of thrombin prevents HEV replication. **(A)** Huh7 cells were electroporated with the WT HEV replicon RNA or GNN control replicon before the addition of AEBSF (2.5 μg / mL), antipain (50 μg / mL), chymostatin (25 μg / mL), E-64 (100 μg / mL) or leupeptin (175 μg / mL) 24 h post-electroporation. Cells were harvested 96 hours post-electroporation and luciferase activity determined. Data shown represents log_10_ of mean relative luciferase activity at 96 h post-electroporation (n = 3 +/- SEM, * = p<0.05, ** = p<0.01 compared to untreated). **(B)** BV-2 cells were infected with MNV at a MOI of 0.1 before the addition of AEBSF (2.5 μg / mL), antipain (50 μg / mL), chymostatin (25 μg / mL), E-64 (100 μg / mL) or leupeptin (175 μg / mL). Virus replication was determined by TCID_50_ assay 16 hours post-infection on naïve BV-2 cells. Data shows mean TCID_50_ / mL (n = 3 +/- SEM, *** = p<0.001 compared to untreated). **(C)** Huh7 cells were electroporated with the WT HEV replicon RNA or GNN control replicon before the addition of apixiban (42 μM), dabigatran (42 μM), warfarin (830 μM) or DMSO solvent control (untreated) 24 h post-electroporation. Cells were harvested 96 h post-electroporation and luciferase activity determined. Data shown represents log_10_ of mean relative luciferase activity at 96 h post-electroporation (n = 3 +/- SEM, * = p<0.05, ** = p<0.01 compared to untreated).

The HEV PCP has been suggested to have a cysteine active site or act like a metalloprotease [39–41,43,60]. In contrast, thrombin is a serine protease. This difference allowed us to use a range of commercially available selective protease inhibitors to test inhibition of replicon replication. Five protease inhibitors were chosen based on their specificity. AEBSF is an irreversible serine protease inhibitor that inhibits chymotrypsin-like proteases including trypsin and thrombin. E-64 is an irreversible cysteine protease inhibitor that includes papain-like proteases. Leupeptin is an inhibitor which can target a range of proteases including cysteine, serine and threonine proteases, including trypsin and papain, but importantly has lower specificity for thrombin. Antipain is a reversible serine/cysteine protease inhibitor of broad spectrum with a similar action to leupeptin, but which includes thrombin. Chymostatin is an inhibitor of many proteases, including chymotrypsin and papain as well as chymotrypsin-like serine proteinases. A single concentration of each inhibitor was chosen based on the maximal tolerated concentration (S7 Fig). Huh7 cells were electroporated with the WT G1 replicon or GNN control prior to the addition of a range of protease inhibitors at 24 h post-electroporation and monitoring of luciferase activity over four days to measure RNA replication (Figs 6A and S7). As a control the panel of inhibitors were tested for effect on MNV, which has a very well characterised cysteine protease [61]. In this case viral replication was assayed by measuring production of infectious virus by TCID_50_ assay after treatment with the panel of inhibitors (Fig 6B).

Upon treatment with this range of inhibitors, only AEBSF and antipain significantly inhibited replicon replication, reducing luciferase activity ~10-fold and ~3-fold at 4 days post-electroporation, respectively. Similar reductions in replication were also observed at earlier time-points in the experiment. The other inhibitors tested did not change luciferase activity compared to the untreated control at any time-point tested (Figs 6A and S7). In contrast, both E-64 and leupeptin significant reduced virus replication by ~10-fold compared to the untreated control (Fig 6B). Neither AEBSF nor antipain reduced MNV replication throughout the duration of the experiment, suggesting specificity for HEV replication.

Based on the differences in inhibition between leupeptin and antipain we went on to specifically target thrombin using a series of well characterised and clinically-used coagulation inhibitors. Dabigitran directly and reversibly binds to the active site of thrombin. Warfarin blocks vitamin K epoxide reductase (required for reactivation of vitamin K1), which is required for production of several clotting factors including prothrombin. Alongside we also tested apixaban, a direct reversible inhibitor of factor Xa, another serine protease clotting enzyme that is produced in hepatocytes but with a different cleavage specificity. Again, a single concentration of each inhibitor was chosen based on the maximal tolerated concentration (S7 Fig). Huh7 cells were therefore electroporated with the WT G1 prior to the addition of these protease inhibitors at 24 h post-electroporation and monitoring of luciferase activity over four days to measure RNA replication (Figs 6C and S7).

Both dabigatran and warfarin significantly inhibited replicon replication, reducing luciferase activity by ~10-fold and ~4-fold compared to untreated, respectively, at 4 days post-electroporation (Fig 6C). Significant reductions in replication were also observed at both 2 and 3 days post-electroporation (S7 Fig). Apixaban did not reduce luciferase activity at any time-point tested. In comparison to cytotoxicity (S7 Fig), AEBSF and dabigatran were the only inhibitors to display cytotoxicity at any of the concentrations tested after 3 days of treatment. However, no significant cytotoxicity was observed with any of the inhibitors with the concentration used in the HEV replication assays. Taken together, the results from the replicon replication assays suggest thrombin is an important host factor for HEV replication.

### Thrombin is generated in HEV electroporated cells

**Fig 7:**
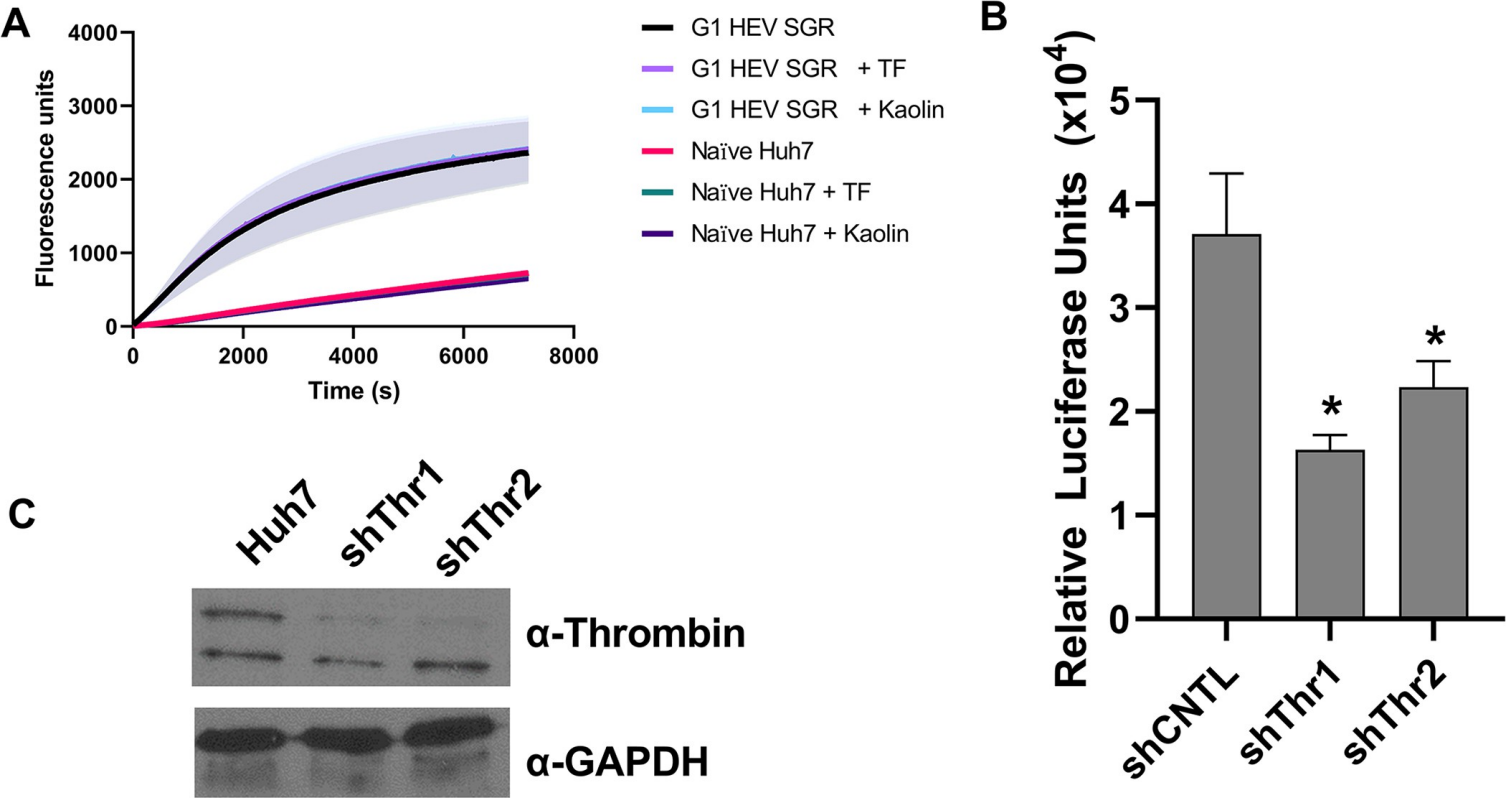
Active thrombin is produced in Huh7 cells. **(A)** Huh7 cells were electroporated with the G1 nLuc SGR or mock electroporated. Four days post-electroporation cells were lysed and post-nuclear supernatant used for thrombin generation assays (n = 3 +/- SEM). **(B)** Huh7 cells silenced for thrombin by shRNA (shThr) or a scramble shRNA control (shCNTL) were electroporated with WT G1 nLuc SGR RNA. Cells were harvested regular intervals post-electroporation and luciferase activity determined. Data shown represents log_10_ of mean relative luciferase activity at 96 h post-electroporation (n = 3 +/- SEM, * = p<0.05 compared to shCNTL). **(C)** Western blot showing silencing of thrombin in shThr1 and shThr2 cell lines.

Next, we sought to try and detect the production of active thrombin within Huh7 cells or Huh7 cells electroporated with a HEV SGR. Huh7 cells were therefore electroporated with the WT G1 HEV SGR RNA or mock electroporated. Four days post-electroporation total cell lysate was prepared by hypotonic lysis and nuclei removed to acquire a total cell post-nuclear supernatant. This supernatant was used in a fluorescent thrombin generation assay (modified calibrated automated thrombogram) and in a turbidity assays of thrombin-mediated fibrinogen to fibrin conversion to detect the presence of active thrombin (Figs 7A and S8A).

In thrombin generation assays it was clear that the post-nuclear supernatant from cells electroporated with G1 HEV SGR contained high concentrations of already active thrombin that cleaved the thrombin substrate more and at a faster rate than supernatant from naïve Huh7 cells (Fig 7A). Moreover, the addition of Tissue Factor (TF) or Kaolin did not result in extra active thrombin being produced in either the G1 HEV SGR or naïve Huh7 cells. This suggests that extra prothrombin cannot be activated through the intrinsic or extrinsic coagulation cascade and that HEV may be able to activate prothrombin through different mechanisms.

Turbidity experiments demonstrated that the thrombin produced in transfected cells was “normal” and functional as it was able to convert purified fibrinogen to fibrin, allowing fibrin polymerisation and the formation of a fibrin clot (S8A Fig). Trace amounts of thrombin were detected in the supernatant from naïve Huh7 cells in both the thrombin generation with low cleavage of the thrombin substrate and turbidity experiments with some fibrin formation, however this was at very low levels.

The previous report by Kanade *et al*, also suggested that silencing of thrombin by siRNA was able to reduce replication of a *Renilla* luciferase Sar55 G1 replicon by ~50% [47]. To confirm those observations, we generated two Huh7 cells lines where thrombin was silenced using lentivirus expressing two different thrombin shRNAs (Fig 7B and 7C). In agreement with the previous study, both thrombin shRNA silenced cell lines (shThr1 and shThr2) significantly reduced replication of the G1 SGR by approximately 50% compared to the scramble control (shCNTL).

### Partial co-localisation between thrombin and pORF1 proteins

For thrombin to process pORF1 there would likely be co-localisation between these two proteins within the cell. This co-localisation is also likely to be partial or transient to allow time for proteolysis before the pORF1 products can form sites of replication. The lack of available well-characterised pORF1 antibodies has hampered many immunofluorescence studies, until recently when it was identified that tolerated insertion sites were located within the HVR region of a G3 virus [46]. Based on these studies we adapted the G1 nLuc SGR in our study to insert a V5 epitope tag in the HVR either after amino acid 731 or 744 of pORF1 (termed G1-731^V5^ and G1-744^V5^, respectively). To confirm replication competency, RNA from these replicons was electroporated into Huh7 cells along with a wild-type (WT) control replicon or the replicon-defective control (GNN), and luciferase activity was monitored over four days to measure RNA replication (S8B Fig). For the G1-731^V5^ SGR, luciferase activity was ~100-fold higher than the replication-defective control (GNN), demonstrating that insertion of the V5 sequence did not prevent replication. However, in comparison to the WT control there was a ~7-fold reduction in replication but this was not significant. In contrast the G1-744^V5^ replicon was unable to replicate suggesting insertion of the V5 tag at this location was incompatible with genome replication.

**Fig 8:**
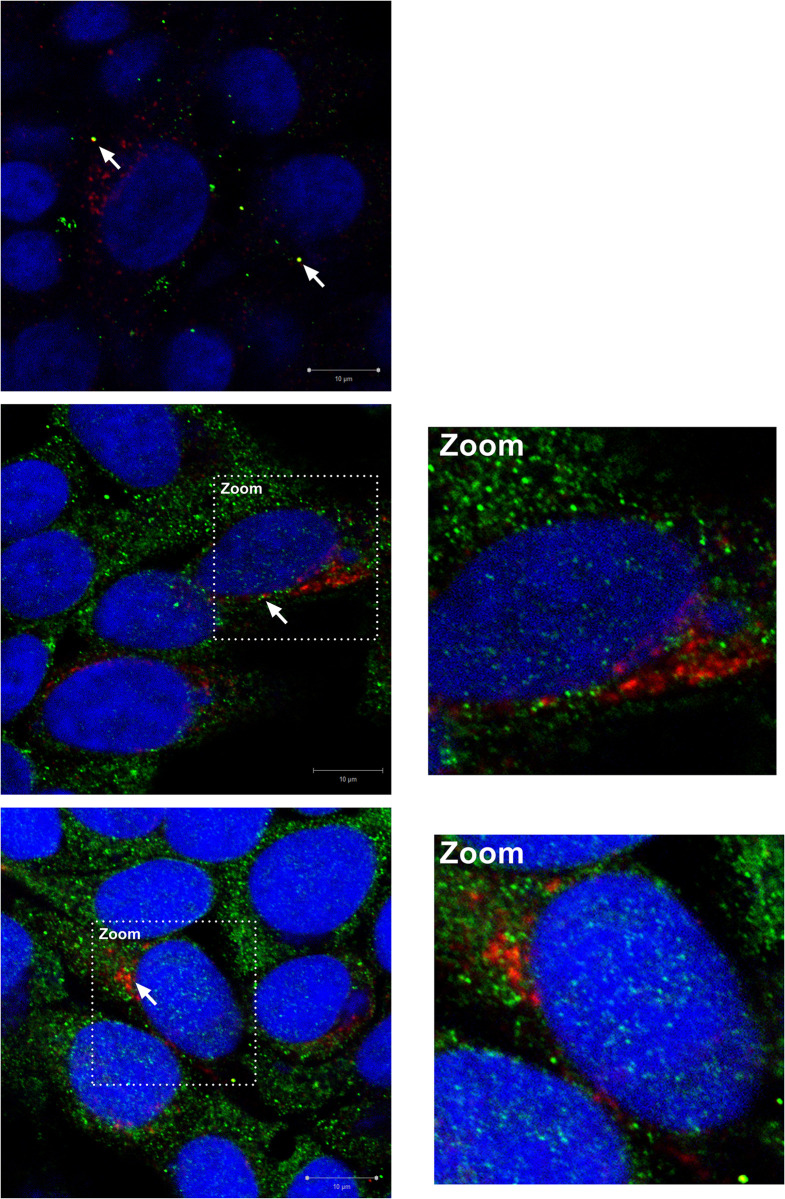
Co-immunofluorescence of epitope tagged pORF1 and (pro)thrombin. Huh7 cells were electroporated with G1-731^V5^ RNA, fixed at 96 h post-electroporation and labelled using anti-thrombin (green) and anti-V5 (red) antibodies. Cell nuclei were stained with NucBlue (blue) and images captured on a Zeiss LSM-880 confocal microscope under oil immersion at 63x magnification (bar 10 μm). White arrows depict areas of co-localisation of close association between G1-731^V5^ and thrombin.

Having confirmed replication competency of the G1-731^V5^ SGR, this RNA was electroporated into Huh7 cells and co-localisation of thrombin and/or prothrombin and V5-tagged pORF1 imaged by anti-body labelling and co-immunofluorescence at 4 days post-electroporation (Fig 8).

The anti-(pro)thrombin antibody detected a broad distribution of (pro)thrombin in foci through the cell cytoplasm. Cells expressing G1-731^V5^ showed expression of pORF1 in the cytoplasm as many distinct foci or puncta, similar to that previously observed but without any nuclear localisation as has been observed in some studies using different expression systems [17,42,46]. There was a high degree of co-localisation between (pro)thrombin and G1-731^V5^ in some distinct foci within the cells, however, this co-localisation was in the minority (Manders coefficient 0.553 +/- 0.122 S.D. of 731^V5^ overlapping with thrombin). Some G1-731^V5^ foci were also in close association with areas of (pro)thrombin expression. However, the majority of G1-731^V5^ did not co-localise to (pro)thrombin within the cell suggesting any interaction is partial or transient (Manders coefficient 0.403 +/- 0.339 S.D. of thrombin overlapping with 731^V5^).

## Discussion

Positive-sense RNA viruses in general encode polyproteins that undergo precise proteolysis to generate functional units referred to as non-structural (NS) proteins. These NS proteins assemble into active genome replication complexes also termed the replicase. Insight into viral polyprotein processing is therefore important for understanding how the replication complex is formed and the functional proteins within this.

Multiple studies have attempted to understand processing of the HEV polyprotein (pORF1), providing some data or suggestions on how this may occur. The results from these studies can be divided into two broadly conflicting models. Several reports have provided evidence for pORF1 processing, generating either two larger products of ~110 and ~80 kDa, multiple products ranging in size from ~18 to ~120 kDa [36–41]. In contrast, data from several studies suggest that pORF1 is not processed and could function as a single polyprotein [46,62,63]. This is supported by the observations that no protease activity has been attributed to the postulated viral protease, PCP [43,44]. The reasons behind the contradictions in these studies are not clear but is possibly due to the wide range of expression systems (i.e., *in vitro* transcription/translation, insect cells, vaccinia expression, and various mammalian cell lines) and methods (i.e., western blot with custom generated antibodies and/or radiolabelling) used for detection. This variability is emphasised by a recent study comparing processing of a G3 HEV pORF1 in three different heterogeneous expression system. In all systems the main product formed was attributed to be full length pORF1, however, a range of less-abundant processed products were observed between ~95 and ~170 kDa. Most of these were not conserved between systems but there were some common products potentially at ~50, ~120 and ~100 kDa [42].

Here, we found that the pORF1 polyprotein was unable to be processed auto-catalytically using a well-established *in vitro* transcription and translation system, in agreement with studies suggesting pORF1 cannot undergo proteolysis [17,46]. We, and others, have used this system to study the processing of many positive-sense RNA viruses. For example, MNV ORF1 can undergo auto-catalytic processing in this system to generate the same range of proteins found during natural infection [50,56,57]. To attempt to stimulate intrinsic protease activity of pORF1 in this system we supplemented the reactions with various metal ions, reducing agents, fatty acids and cellular extracts. Despite this, none of these factors could elicit protease activity and change the products formed. In addition, we mutated eight of the amino acids suggested to be the protease active site [39–41,43], however, again none of the mutants changes the products formed. All these observations support the previous studies suggesting that pORF1 has no auto-catalytic activity, and that the PCP domain does not have protease activity.

**Fig 9:**
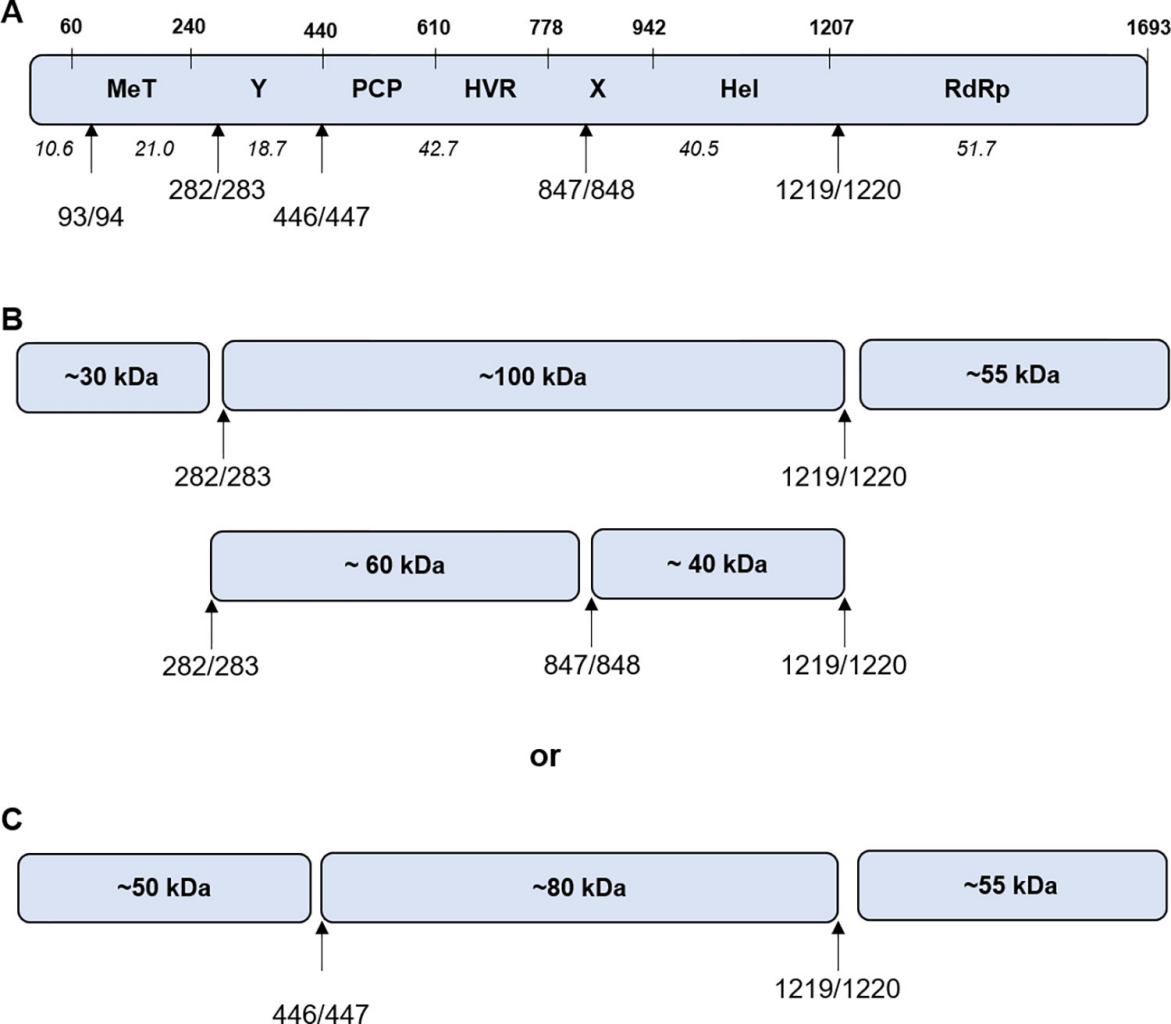
Schematic of thrombin-mediated proteolysis of pORF1. **(A)** Schematic of G1 pORF1 showing the putative thrombin cleavage sites investigated in this study. Numbers in italics show the predicted molecular weight of products after theoretical complete thrombin proteolysis. **(B)** The observed ~ 30, ~100 and ~55 kDa products would be explained by processing at the PR282/283 and PR1219/1220 position. Subsequent processing at PR847/848 would yield ~60 and ~40 kDa products. **(C)** The observed ~ 50, ~80 and ~55 kDa products would be explained by processing at the PR446/447 and PR1219/1220 position.

In addition to this work, a single study by Kanade *et al*, implicated two host proteases, thrombin and factor Xa, as important for HEV G1 replication [47]. They showed that these enzymes were able to cleave the purified fragments of pORF1 at two places and siRNA silencing of thrombin expression reduced HEV replication. The authors therefore suggested that thrombin was important for HEV replication. By comparative alignment of HEV sequences, we identified additional sites that match the thrombin recognition sequence [53], six of which were highly conserved (S1 Fig). Indeed, we found that addition of exogenous thrombin to the *in vitro* transcription/translation assays processed both the G1 and G3 pORF1 polyprotein into at least 9 similar products. Using a combination of mutagenesis and polyprotein truncations we demonstrated that six of these seven sites were cleavable by thrombin *in vitro* and prevented G1 and G3 replicon replication. Interestingly, these were sites of high sequence conservation across HEV isolates. The remaining site (PR638/639 for G1 and PR757/758 for G3, respectively) was poorly conserved and only reduced replication ~2-fold when mutated in a G1 replicon (which had low homology to the thrombin cleavage consensus) but completely prevented replication of a G3 replicon. These data would suggest therefore that the PR638/639 site may not be a genuine processing site, or at least these residues are of variable importance in replication of different HEV genotypes. Given that we observed at least nine products (excluding full-length pORF1), yet there are only 6 cleavage sites, some of the products observed must be polyprotein precursors. To help us theoretically assign the nine observed products to different cleavage events, we compared the products from proteolysis of the full length G1 pORF1 to the truncated regions (Fig 9). Based on molecular weight, it is likely that products at ~100 to ~60 kDa were large precursors spanning multiple domains. For example, a ~100 kDa product is possible the result of cleavage at the G1 positions PR282/283 and PR1219/1220, whereas products at ~80 kDa could be result of processing at PR446/447 and PR1219/1220 with G1. Processing at PR1219/1220 would yield a ~55 kDa product that would include the viral RdRp. This product was also immunoprecipitated from the reaction using C-terminally epitope tagged constructs, supporting this identification. It is also clear that the ~50 kDa product and doublet at ~30 kDa are only present at the N-terminal portion of the polyprotein and likely be the result of cleavage at the PR446/447 and PR282/283 positions, respectively. The larger products could undergo further proteolysis to generate many of the same final products, for example, the ~40 kDa product found within the C-terminus is likely to be the result of thrombin proteolysis at PR847/848 and PR1219/1220 and could be derived from either ~100 or ~80 kDa precursors. Although, it is challenging to directly compare the processing results from this study to those previous studies where processing was observed, in part due to variations in systems/sequence used, some of the larger molecular weight produce we observed could be consistent with those observed with G1 and G3 heterogeneous expression system. For example, Metzger *et al* also observed a ~100 kDa product with G3 pORF1 across three different expression system [42].

It is not clear from the data presented here whether thrombin is responsible for HEV pORF1 processing in infected cells to generate functional replicase proteins. It is also possible thrombin could work in conjunction with other host proteases or another related serine protease may be responsible for pORF1 proteolysis (e.g., hepsin in the endoplasmic reticulum). Furthermore, several recent studies have used *in silico* modelling to predict potential pORF1 domain structures and macromolecular assemblies [16–18]. The position of some thrombin cleavage junctions would be compatible with some of these predictions, for example the PR1229/1220 junction that could release RdRp. However, other junctions such as those at PR282/283 could disrupt the suggested Met capping pore formation [18]. Further work is therefore needed to conclusively confirm the role of thrombin and the identity of all pORF1 products in cells.

If thrombin is solely responsible for the processing of pORF1 it would represent a new mechanism of polyprotein cleavage. Thrombin is synthesised specifically in hepatocytes as the inactive complex multi-domain zymogen prothrombin [49,64]. Prothrombin is secreted into the blood where the Gla and kringle domains are removed by enzymes in the prothrombinase complex to generate the active enzyme thrombin (via intermediates) in the clotting cascade [48,65]. However, several reports have identified active thrombin at detectable levels in hepatocytes where it is believed to play some role in cancer regulation [66–68]. Indeed, we also found that HEV replicon replication was sensitive to thrombin down-regulation, serine and not cysteine protease inhibitors and clinically used thrombin (but not factor Xa) inhibitors, suggesting an important role for thrombin in HEV replicon replication. Active thrombin could be detected in Huh7 cells in very low concentrations by immunoblot and thrombin generation assays. However, electroporation of a HEV G1 replicon into Huh7 cells significantly increased thrombin generation 96 hours after electroporation, and the thrombin produced was unlikely to be generated through a Xa/coagulation cascade mechanism. These data suggest that thrombin could therefore be available in infected cells at sufficient concentrations to be functional. However, the low degree of colocalization between pORF1 and thrombin suggests either a transient interaction or no direct role of thrombin in pORF1 processing.

Irrespective of mechanism, if thrombin is essential for HEV genome replication it has important implications for viral zoonosis. Viruses that are able to replicate across species must overcome host cell restriction factors. Thrombin is common to all mammals and is genetically, structurally, and functionally similar across human, bovine and porcine species [69,70]. It could therefore be an advantage for viral transmission to rely on a key and conserved host enzyme. However, this could open up new avenues for novel therapeutic design including the re-design or repurposing of clinically used inhibitors.

## Materials and methods

### Cell lines and plasmids

Huh7 and BV-2 cells (both ATCC, USA) were maintained in Dulbecco’s modified Eagle’s medium with glutamine (Sigma-Aldrich, USA) supplemented with 10% (v/v) FCS, 1% (v/v) non-essential amino acids, 10 mM HEPES, 50 U / mL penicillin and 50 μg / mL streptomycin.

Plasmid carrying wild-type genotype 1 HEV (Sar55 strain; GenBank accession no AF444002) replicon expressing GFP, pSK-E2-GFP, was a kind gift from Dr Patrizia Farci (NIAID, Maryland, USA) and has been described previously [71]. This plasmid was modified to replace the GFP open reading frame with nano-luciferase to generate pSK-E2-nLuc as previously described [72]. The plasmid carrying wild-type genotype 3 HEV (G3-HEV83-2-27 strain; GenBank accession no AB740232) replicon expressing nano-luciferase, pUC-HEV83-2, was a kind gift from Dr Koji Ishii (NIID, Japan) and has been described previously [73]. Mutations within these plasmids were performed by standard two-step overlapping PCR mutagenesis. A negative control pSK-E2-nLuc replicon was generated containing a double point mutation in the RdRp active site GDD motif (GNN). A negative control pUC-HEV83-2 replicon was generated containing a frame-shift in the RdRp protein by digestion with *Kpn*I and Mung Bean nuclease (NEB, USA) followed by re-ligation.

For coupled *in vitro* transcription and translation experiments, pcDNA3.1(+) based expression plasmids were generated by PCR. Firstly, to facilitate cloning a *Not*I restriction enzyme site within the HEV pORF1 coding region was removed by silent mutagenesis. Subsequently, the relevant HEV sequence was amplified to including flanking *Not*I restriction enzymes and upstream Kozak modified translational start site. To insert a HA-epitope at the C-terminus of pORF1 the reverse PCR primer contained a HA sequence before the stop codon. The *Not*I-digested PCR products were cloned into *Not*I digested pcDNA3.1(+) (Thermo Fisher Scientific, USA). The sequence of all primers and plasmids are available on request.

### Coupled transcription and translation reactions

Coupled *in vitro* transcription and translation assays were performed using the TNT Quick Coupled Transcription/Translation system (Promega, USA) following manufacturer’s instruction. Reactions contained 10 μL rabbit reticulocyte lysate with 500 ng of pcDNA T7 expression plasmid and 0.5 μL [^35^S] methionine (PerkinElmer, USA). Reactions were incubated at 30°C for 40 minutes before being chased with 2 μl of 50 mg / mL unlabelled methionine. Plasma-purified human thrombin (Merck Life Sciences, USA) was then added to reactions as required from a 1 IU / μL stock. Reactions were stopped at regular intervals by the addition of 2 x Laemmli buffer. Samples were separated by SDS-PAGE before visualisation of radiolabelled products by CL-Xposure film autoradiography and PhosphorImaging on a PhosphorScreen imaged on a Fujifilm FLA-5100 (Fuji, Japan). High-resolution digital PhoshorScreen images were used to quantify the relative proportions of each product by densitometry as a percentage of total [^35^S] incorporation using ImageJ software.

### *In vitro* transcription

The G1 and G3 HEV replicon plasmids were linearised with *Bgl*II and *Hind*III, respectively, before being used to generate T7 *in vitro* transcribed RNA using the HiScribe T7 ARCA mRNA kit with tailing following manufacturer’s instructions (Promega, USA). RNA was purified using an RNA clean and concentrate kit (Zymo Research, USA) and the quality was checked using MOPS/formaldehyde agarose gel electrophoresis.

### Replication assays

Replicon experiments were conducted as previously described [72]. Briefly, Huh7 cells were detached by trypsin, washed twice in ice-cold DEPC-treated PBS and re-suspended at 1 x 10^7^ cells / mL in DEPC-treated PBS. Subsequently 400 μL of cells was mixed with 2 μg of RNA transcript, transferred to a 4 mm gap electroporation cuvette (SLS, UK) and pulsed at 260 V, 25 ms pulse length in a Bio-Rad Gene Pulser (Bio-Rad, USA). Electroporated cells were recovered into 4 mL media, seeded into replicate 6-well tissue culture vessels, and replication measured at 24 h intervals using Nano-Glo luciferase assay system (Promega, USA). For inhibitor treatment the electroporated cells were seeded into replicate 24-well plates, allowed to adhere for 24 h before the media was replaced with fresh media containing antipain, AEBSF, leupeptin, (Sigma-Aldrich, USA), warfarin, dabigatran etexilate (Merck Life Sciences, USA) or apixiban (Insight Biotechnology, UK), at the indicated concentrations. The cell viability experiments were conducted by seeding cells into 96-well plates, allowing to adhere for 24 h before addition of a serial dilution of protease inhibitors and measurement of cell viability 72 h later using the CellTiter AQueous One solution (Promega, USA), following manufacturer’s instructions.

### Immunoprecipitation

Immunoprecipitation reactions were performed using Dynabeads Protein G (Thermo Fisher Scientific, USA). To bind the antibody to magnetic beads, 10 μL of the anti-HA rabbit antibody (Sigma-Aldrich, USA) was mixed with 195 μL PBS and incubated at room temperature with 50 μL magnetic beads, shaking for 1 h, after which the supernatant was removed from the beads. Transcription and translation reaction samples were mixed with 200 μL PBS and incubated shaking at room temperature with 25 μL of Dynabeads as a pre-clear step. The tube was placed on the magnet and the supernatant removed and added to the 50 μL of Dynabeads with the antibody bound and incubated at room temperature shaking for 1 h. The flow through was removed and added to 2x Laemmli buffer. The beads were washed three times with PBS with 0.02% Tween-20 and each wash supernatant retained. Proteins were eluted from the beads by adding 50 μL of 2x Laemmli buffer and heating to 100°C.

### Immunofluorescence

Huh7 cells were electroporated with G1 replicon RNA containing V5-epitope inserted in the HVR before seeding onto coverslips. At 4 days post-electroporation cells were fixed in 4% paraformaldehyde and washed with PBS prior to immunofluoreescent labelling as previous described [56]. Primary antibodies used were rabbit anti-thombin (Proteintech, USA), which can detect both thrombin and prothrombin, and mouse anti-V5 (Thermo Fisher Scientific, USA) detected with secondary antibodies anti-rabbit Alexa488 and anti-mouse Alexa568 (both Thermo Fisher Scientific, USA), respectively. Coverslips were washed a final time in PDS before mounting in ProLong Glass with NucBlue (Thermo Fisher Scientific, USA). Images were captured using a Zeiss LSM-880 confocal microscope. Manders coefficients were calculated using the JACoP function in ImageJ on ten separate images from which mean and standard deviation values were calculated.

### Lentivirus production and generating silenced cell lines

Huh7 cells silenced for thrombin were produced as previously described [74]. Briefly, HEK293T cells were prepared for transfection in 10 cm dishes. Lentivirus production was achieved via transfection into HEK293T cells of 1 μg p8.9 (packaging plasmid) 1 μg pMDG (VSVg envelope plasmid) and 1.5 μg pHIV-SIREN encoding thrombin shRNA (genome plasmid). Supernatants were harvested at 48 h and 72 h post-transfection and filtered.

Huh7 cells were plated at a density of 1 x 10^5^ cells / well in a 6-well plate. Cells were then transduced with 1 mL / well of lentivirus supernatant in the presence of 8 μg / mL polybrene to promote transduction. Selection with 2.5 μg / mL puromycin was introduced 72 h post-transduction. Passage of cells in puromycin selection media was continued to maintain expression of shRNA.

### Thrombin generation assays

For thrombin generation assays, post-nuclear supernatant from Huh7 cell lysates were prepared using the Endoplasmic Reticulum Isolation kit (Sigma-Aldrich, USA), following manufacturer’s instructions. Briefly, Huh7 cells were electroporated with G1 replicon RNA or no RNA controls, seeded into T175 flasks and cultured for four days before preparation of lysate.

Thrombin generation was measured using a calibrated automated thrombogram system based on the method of Hemker et al [75]. Reagents were purchased from Stago. Tissue factor (1pM) and STA-PTT automate 5 (Kaolin) were used to trigger the extrinsic and intrinsic pathways of coagulation in post-nuclear supernatants. Transfected and un-transfected cell lysates were diluted by half in HBS and loaded onto round-bottom 96-well plates in triplicates. Thrombin calibrator (690 nM activity) was added to calibrator wells, and HBS, Tissue factor or STA-PPT automate was added to the experimental wells. Thrombin generation was initiated by the addition of fluorogenic substrate Z-Gly-Gly-Arg-aminomethylcoumarin and 16.7 mM of CaCl_2_. Fluorescence measurements were obtained every 20 seconds for 120 minutes using a Fluoroscan Ascent reader (Thermo Fisher Scientific, USA). Data obtained were analyzed by the dedicated Hemker Thrombinoscope software (Stago, France). Experiments were carried out in technical triplicate with three biological repeats of lysate preparation.

### Turbidity assays

Fibrin clot formation was analysed by turbidity assays as described using a purified system [76]. Plasma purified fibrinogen was diluted with HBS for a final fibrinogen concentration of 1 mg/ml. This was added to a 96-well Greiner bio-one microtiter plate (Greiner Bio One International GmbH, Austria), and an activation mix added. The activation mix contained CaCl_2_ (10 mM) and either cell lysate from un-transfected or G1 replicon electroporated cells (1/10 dilution) or thrombin (0.5 U / mL). Changes in absorbance at 340 nm was measured every 12 seconds for 2 hours at 37°C in a Multiscan Go Microplate reader (Thermo Fisher Scientific, USA). Experiments were carried out in technical triplicate with three biological repeats of lysate preparation.

### TCID_50_ assays

MNV replication was determined in the presence of inhibitors using a TCID_50_ assay modified from Hwang et al [77]. Briefly, BV-2 cells were seeded into 6 well plates at 2 x 10^5^ cells/well and left overnight. Cells were then infected with MNV-1 at an MOI of 0.1 for 16 hours with or without the presence of antipain, AEBSF, chymostatin, E-64 or leupeptin (as described above) at the indicated concentrations. After 16 hours, supernatant was collected and used to infect BV-2 cells in 96 well plates at 2 x 10^4^ cells/well by serial dilution. TCID_50_ values were calculated according to the Spearman and Kärber algorithm.

## Supporting information

S1 Fig Alignment of the conserved pORF1 thrombin recognition sites. Sequence logos show the conservation of amino acids at the predicted thrombin cleavage junctions across 2114 pORF1 *Hepeviridae* sequence (including 621 complete pORF1 protein sequences) that showed the greatest identity to the Sar55 pORF1 sequence. All numbers correspond to the amino acid positions in the Sar55 sequence (GenBank accession no. AF444002).
(DOCX)

S2 Fig Thrombin proteolysis of the C-terminal portion of pORF1. **(A)** and **(C)** Plasmids expressing C-terminal portions of the pORF1 polyprotein were used to template *in vitro* coupled transcription/translation reactions labelled with [^35^S] methionine without thrombin. Protein samples were taken at the indicated time-points, stopped by the addition of Laemmli buffer, proteins separated by SDS-PAGE and visualised by autoradiography and phosphorimaging. The approximate molecular weight of each product is indicated together with the molecular weight ladder in kDa on the left of each gel. The proportion of each product in Fig 1 (C) and (E) was quantified as a percentage of total [^35^S] incorporation and is shown in **(B)** and **(D)**, respectively (n = 2 +/- SD).
(DOCX)

S3 Fig Thrombin proteolysis of the N-terminal portion of pORF1. **(A)** Schematic of the truncated pORF1 expression plasmid. **(B)** A plasmid expressing the N-terminal portion of the WT pORF1 polyprotein were used to template *in vitro* coupled transcription/translation reactions labelled with [^35^S] methionine. **(C-G)** Plasmid expressing amino acids 1–712 of pORF1 with the indicated alanine substitutions at amino acids **(C)** PR52/53, **(D)** PR93/94, **(E)** PR282/283, **(F)** PR446/447, **(G)** PR638/639, before being used to template [^35^S] methionine labelled *in vitro* coupled transcription/translation reactions. Where indicated to duplicate reactions a zero minute sample was taken before the addition of 0.5 IU of thrombin followed by collection of protein samples at the indicated time-points representing minutes after the addition of thrombin. Samples were harvested into Laemmli buffer to stop reactions, proteins separated by SDS-PAGE and visualised by autoradiography and phosphorimaging. The approximate molecular weight of each product is indicated together with the molecular weight ladder on the left of the gel (n = 2 +/- SD).
(DOCX)

S4 Fig Thrombin proteolysis of the N-terminal portion of pORF1. Site directed mutagenesis was used to introduce alanine substitutions at either the PR53/54, PR93/94, PR282/283, PR446/447 or PR638/639 residue pairs within the context of the 1–712 precursor. These plasmids were used to template *in vitro* coupled transcription/translation reactions labelled with [^35^S] methionine before the addition of 0.5 IU of thrombin. Proteins were separated by SDS-PAGE and visualised by autoradiography (shown in Fig 2). The relative proportions of the **(A)** ~80 kDa, **(B)** ~70 kDa, **(C)** ~40 kDa and **(D)** ~30 kDa proteins were quantified from each of these substitutions in comparison to the WT control (n = 2 +/- SD).
(DOCX)

S5 Fig Thrombin proteolysis of pORF1. **(A)** Plasmids G1 HEV pORF1 were used to template *in vitro* coupled transcription/translation reactions labelled with [^35^S] methionine. Samples were taken at regular intervals, reactions stopped by the addition of Laemmli buffer, proteins separated by SDS-PAGE and visualised by autoradiography and phosphorimaging. To a reaction with HEV G1 pORF1 or G3 pORF1 0.5 IU thrombin was added as indicated and product of thrombin proteolysis indicated together with their approximate molecular weight (shown in Fig 3). **(B)** The relative portion of each product for G1 pORF1 was quantified as a percentage of the total [^35^S] incorporation (n = 2 +/- SD; * = p<0.05, ** = p<0.01, *** = p<0.001).
(DOCX)

S6 Fig Preventing thrombin proteolysis prevents HEV replication. Huh7 cells were electroporated with **(A)** G1 or **(B)** G3 HEV replicon RNA containing the indicated mutations at predicted thrombin cleavage junctions, in addition to the WT and GNN control replicons. Cells were harvested at the indicated times post-electroporation and luciferase activity determined. Data shown represents log_10_ of mean relative luciferase activity (n = 3 +/- SEM).
(DOCX)

S7 Fig Pharmacological inhibition of thrombin prevents HEV replication. **(A)** Huh7 cells were electroporated with the WT HEV replicon RNA or GNN control replicon before the addition of AEBSF (2.5 μg / mL), antipain (50 μg / mL), chymostatin (25 μg / mL), E-64 (100 μg / mL) or leupeptin (175 μg / mL) 24 h post-electroporation. Cells were harvested at the indicated times post-electroporation and luciferase activity determined. Data shown represents log_10_ of mean relative luciferase activity (n = 3 +/- SEM, * = p<0.05, ** = p<0.01 compared to untreated). **(B)** Huh7 cells were electroporated with the WT HEV replicon RNA or GNN control replicon before the addition of apixiban (42 μM), dabigatran (42 μM), warfarin (830 μM) or DMSO solvent control (untreated) 24 h post-electroporation. Cells were harvested 96 hours post-electroporation and luciferase activity determined. Data shown represents log_10_ of mean relative luciferase activity at 96 h post-electroporation (n = 3 +/- SEM, * = p<0.05, ** = p<0.01 compared to untreated). **(C)** and **(D)** Huh7 cells were incubated with a serial dilution of indicated compounds for 72 hours before cell viability was measured by MTS assay. Data are expressed as mean percentage cell viability normalized to untreated controls (n = 3 +/- SEM).
(DOCX)

S8 Fig Replication of epitope tagged G1 HEV SGR. **(A)** Huh7 cells were electroporated with the G1 nLuc SGR or mock electroporated. Four days post-electroporation cells were lysed and post-nuclear supernatant used in a turbidity assay of thrombin-mediated fibrinogen to fibrin conversion (n = 3 +/- SEM). **(B)** Huh7 cells were electroporated with G1 HEV replicon RNA containing the indicated insertions, in addition to the WT and GNN control replicons. Cells were harvested at the indicated times post-electroporation and luciferase activity determined. Data shown represents log_10_ of mean relative luciferase activity (n = 2 +/- SEM).
(DOCX)
