# Supplementary material for: Thrombin cleavage of the hepatitis E virus polyprotein at multiple conserved locations is required for genome replication
Source: PLoS Pathog. 2023 Jul 21;19(7):e1011529. doi: 10.1371/journal.ppat.1011529 (PMC10395923; doi:10.1371/journal.ppat.1011529)
Supplement: S1 Fig — Sequence logos show the conservation of amino acids at the predicted thrombin cleavage junctions across 2114 pORF1 Hepeviridae sequence (including 621 complete pORF1 protein sequences) that showed the greatest identity to the Sar55 pORF1 sequence. All numbers correspond to the amino acid positions in the Sar55 sequence (GenBank accession no. AF444002). (DOCX) [file ppat.1011529.s001.docx]

**S1 Fig**


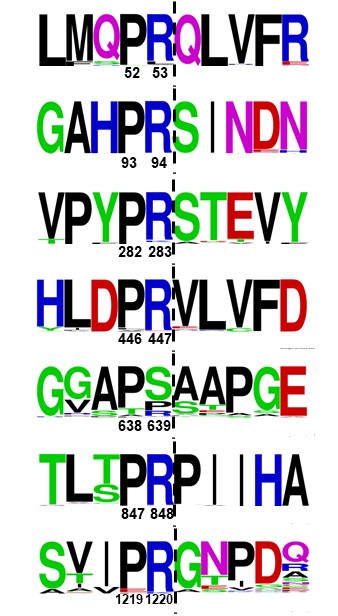


**S1 Fig. Alignment of the conserved pORF1 thrombin recognition sites.** Sequence logos show the conservation of amino acids at the predicted thrombin cleavage junctions across 2114 pORF1 *Hepeviridae* sequence (including 621 complete pORF1 protein sequences) that showed the greatest identity to the Sar55 pORF1 sequence. All numbers correspond to the amino acid positions in the Sar55 sequence (GenBank accession no. AF444002).
