# Supplementary material for: Thrombin cleavage of the hepatitis E virus polyprotein at multiple conserved locations is required for genome replication
Source: PLoS Pathog. 2023 Jul 21;19(7):e1011529. doi: 10.1371/journal.ppat.1011529 (PMC10395923; doi:10.1371/journal.ppat.1011529)
Supplement: S2 Fig — (A) and (C) Plasmids expressing C-terminal portions of the pORF1 polyprotein were used to template in vitro coupled transcription/translation reactions labelled with [35S] methionine without thrombin. Protein samples were taken at the indicated time-points, stopped by the addition of Laemmli buffer, proteins separated by SDS-PAGE and visualised by autoradiography and phosphorimaging. The approximate molecular weight of each product is indicated together with the molecular weight ladder in kDa on the left of each gel. The proportion of each product in Fig 1 (C) and (E) was quantified as a percentage of total [35S] incorporation and is shown in (B) and (D), respectively (n = 2 +/- SD). (DOCX) [file ppat.1011529.s002.docx]

**S2 Fig**


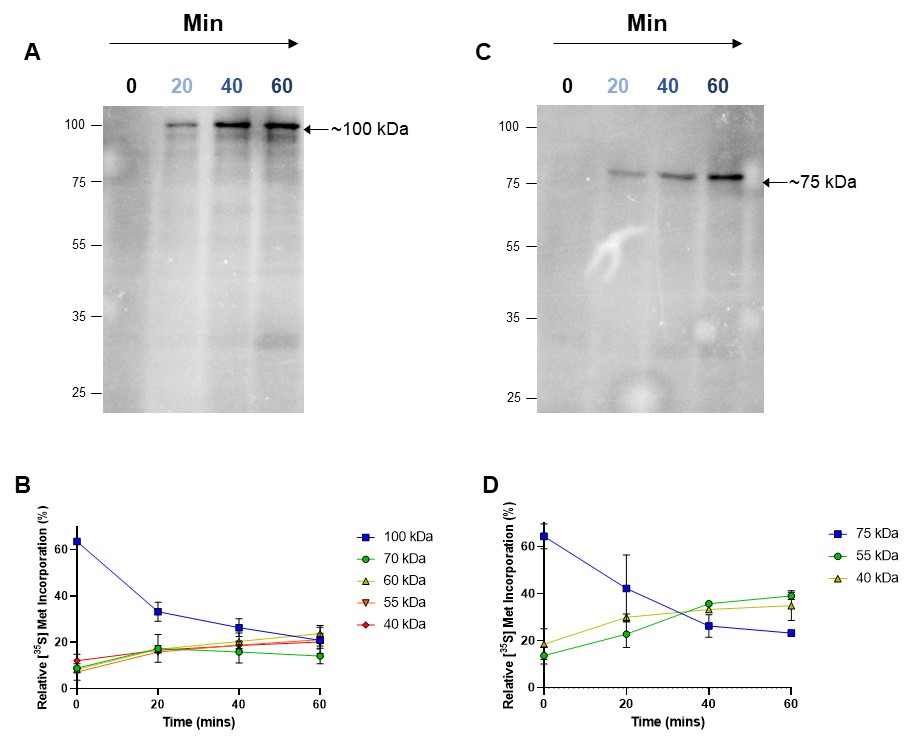


**S2 Fig. Thrombin proteolysis of the C-terminal portion of pORF1. (A)** and **(C)** Plasmids expressing C-terminal portions of the pORF1 polyprotein were used to template *in vitro* coupled transcription/translation reactions labelled with [^35^S] methionine without thrombin. Protein samples were taken at the indicated time-points, stopped by the addition of Laemmli buffer, proteins separated by SDS-PAGE and visualised by autoradiography and phosphorimaging. The approximate molecular weight of each product is indicated together with the molecular weight ladder in kDa on the left of each gel. The proportion of each product in Figure 1 (C) and (E) was quantified as a percentage of total [^35^S] incorporation and is shown in **(B)** and **(D)**, respectively. (n = 2 +/- SD).
