# Supplementary material for: Thrombin cleavage of the hepatitis E virus polyprotein at multiple conserved locations is required for genome replication
Source: PLoS Pathog. 2023 Jul 21;19(7):e1011529. doi: 10.1371/journal.ppat.1011529 (PMC10395923; doi:10.1371/journal.ppat.1011529)
Supplement: S3 Fig — (A) Schematic of the truncated pORF1 expression plasmid. (B) A plasmid expressing the N-terminal portion of the WT pORF1 polyprotein were used to template in vitro coupled transcription/translation reactions labelled with [35S] methionine. (C-G) Plasmid expressing amino acids 1–712 of pORF1 with the indicated alanine substitutions at amino acids (C) PR52/53, (D) PR93/94, (E) PR282/283, (F) PR446/447, (G) PR638/639, before being used to template [35S] methionine labelled in vitro coupled transcription/translation reactions. Where indicated to duplicate reactions a zero minute sample was taken before the addition of 0.5 IU of thrombin followed by collection of protein samples at the indicated time-points representing minutes after the addition of thrombin. Samples were harvested into Laemmli buffer to stop reactions, proteins separated by SDS-PAGE and visualised by autoradiography and phosphorimaging. The approximate molecular weight of each product is indicated together with the molecular weight ladder on the left of the gel (n = 2 +/- SD). (DOCX) [file ppat.1011529.s003.docx]

**S3 Fig**


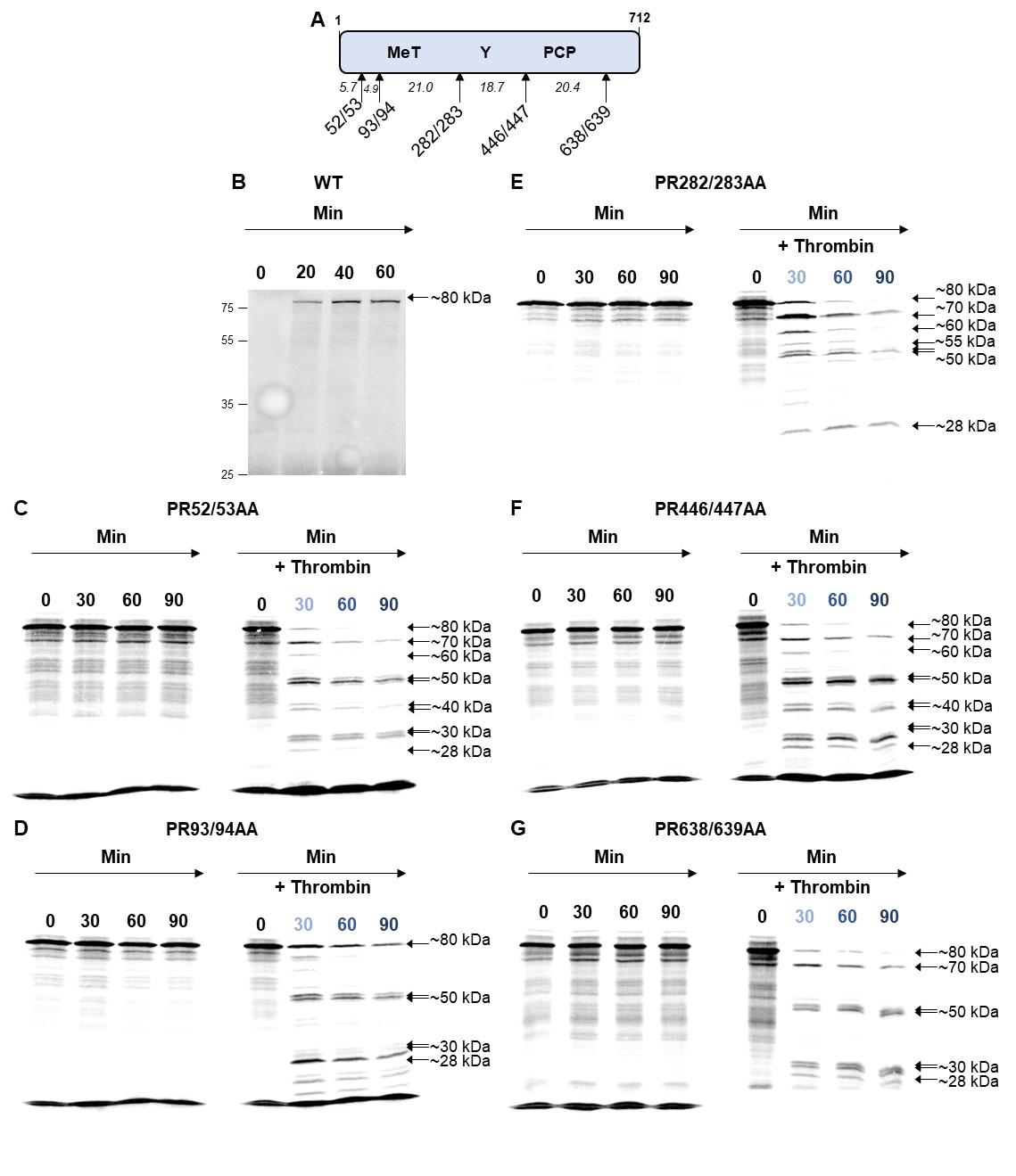


**Figure S3. Thrombin proteolysis of the N-terminal portion of pORF1. (A)** Schematic of the truncated pORF1 expression plasmid. **(B)** A plasmid expressing the N-terminal portion of the WT pORF1 polyprotein were used to template *in vitro* coupled transcription/translation reactions labelled with [^35^S] methionine. **(C-G)** Plasmid expressing amino acids 1-712 of pORF1 with the indicated alanine substitutions at amino acids **(C)** PR52/53, **(D)** PR93/94, **(E)** PR282/283, **(F)** PR446/447, **(G)** PR638/639, before being used to template [^35^S] methionine labelled *in vitro* coupled transcription/translation reactions. Where indicated to duplicate reactions a zero minute sample was taken before the addition of 0.5 IU of thrombin followed by collection of protein samples at the indicated time-points representing minutes after the addition of thrombin. Samples were harvested into Laemmli buffer to stop reactions, proteins separated by SDS-PAGE and visualised by autoradiography and phosphorimaging. The approximate molecular weight of each product is indicated together with the molecular weight ladder on the left of the gel (n = 2 +/- SD).
