# Supplementary material for: Thrombin cleavage of the hepatitis E virus polyprotein at multiple conserved locations is required for genome replication
Source: PLoS Pathog. 2023 Jul 21;19(7):e1011529. doi: 10.1371/journal.ppat.1011529 (PMC10395923; doi:10.1371/journal.ppat.1011529)
Supplement: S4 Fig — Site directed mutagenesis was used to introduce alanine substitutions at either the PR53/54, PR93/94, PR282/283, PR446/447 or PR638/639 residue pairs within the context of the 1–712 precursor. These plasmids were used to template in vitro coupled transcription/translation reactions labelled with [35S] methionine before the addition of 0.5 IU of thrombin. Proteins were separated by SDS-PAGE and visualised by autoradiography (shown in Fig 2). The relative proportions of the (A) ~80 kDa, (B) ~70 kDa, (C) ~40 kDa and (D) ~30 kDa proteins were quantified from each of these substitutions in comparison to the WT control (n = 2 +/- SD). (DOCX) [file ppat.1011529.s004.docx]

**S4 Fig**

**S4 Fig. Thrombin proteolysis of the N-terminal portion of pORF1.** Site directed mutagenesis was used to introduce alanine substitutions at either the PR53/54, PR93/94, PR282/283, PR446/447 or PR638/639 residue pairs within the context of the 1-712 precursor. These plasmids were used to template *in vitro* coupled transcription/translation reactions labelled with [^35^S] methionine before the addition of 0.5 IU of thrombin. Proteins were separated by SDS-PAGE and visualised by autoradiography (shown in Figure 2). The relative proportions of the **(A)** ~80 kDa, **(B)** ~70 kDa, **(C)** ~40 kDa and **(D)** ~30 kDa proteins were quantified from each of these substitutions in comparison to the WT control (n = 2 +/- SD).
