# Supplementary material for: Thrombin cleavage of the hepatitis E virus polyprotein at multiple conserved locations is required for genome replication
Source: PLoS Pathog. 2023 Jul 21;19(7):e1011529. doi: 10.1371/journal.ppat.1011529 (PMC10395923; doi:10.1371/journal.ppat.1011529)
Supplement: S5 Fig — (A) Plasmids G1 HEV pORF1 were used to template in vitro coupled transcription/translation reactions labelled with [35S] methionine. Samples were taken at regular intervals, reactions stopped by the addition of Laemmli buffer, proteins separated by SDS-PAGE and visualised by autoradiography and phosphorimaging. To a reaction with HEV G1 pORF1 or G3 pORF1 0.5 IU thrombin was added as indicated and product of thrombin proteolysis indicated together with their approximate molecular weight (shown in Fig 3). (B) The relative portion of each product for G1 pORF1 was quantified as a percentage of the total [35S] incorporation (n = 2 +/- SD; * = p<0.05, ** = p<0.01, *** = p<0.001). (DOCX) [file ppat.1011529.s005.docx]

**S5 Fig**


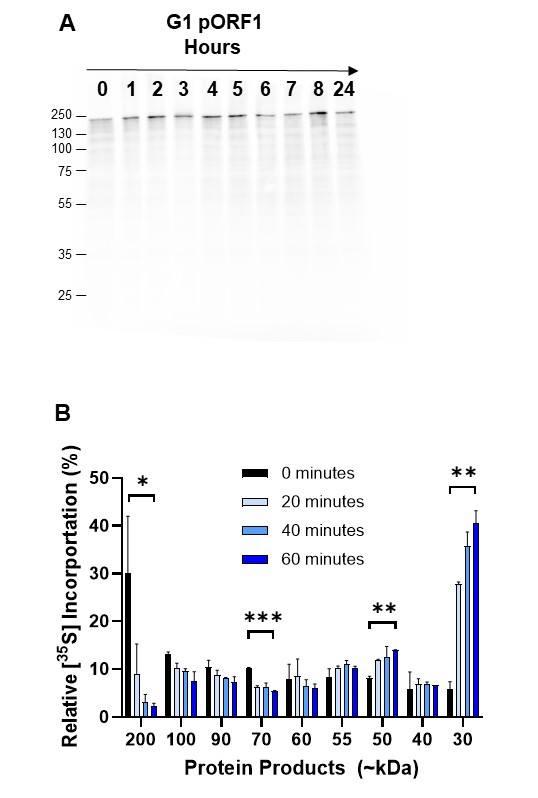


**S5 Fig. Thrombin proteolysis of pORF1. (A)** Plasmids G1 HEV pORF1 were used to template *in vitro* coupled transcription/translation reactions labelled with [^35^S] methionine. Samples were taken at regular intervals, reactions stopped by the addition of Laemmli buffer, proteins separated by SDS-PAGE and visualised by autoradiography and phosphorimaging. To a reaction with HEV G1 pORF1 or G3 pORF1 0.5 IU thrombin was added as indicated and product of thrombin proteolysis indicated together with their approximate molecular weight (shown in Figure 3). **(B)** The relative portion of each product for G1 pORF1 was quantified as a percentage of the total [^35^S] incorporation (n = 2 +/- SD; *=p<0.05, **=p<0.01, ***=p<0.001).
