# Supplementary material for: Thrombin cleavage of the hepatitis E virus polyprotein at multiple conserved locations is required for genome replication
Source: PLoS Pathog. 2023 Jul 21;19(7):e1011529. doi: 10.1371/journal.ppat.1011529 (PMC10395923; doi:10.1371/journal.ppat.1011529)
Supplement: S6 Fig — Huh7 cells were electroporated with (A) G1 or (B) G3 HEV replicon RNA containing the indicated mutations at predicted thrombin cleavage junctions, in addition to the WT and GNN control replicons. Cells were harvested at the indicated times post-electroporation and luciferase activity determined. Data shown represents log10 of mean relative luciferase activity (n = 3 +/- SEM). (DOCX) [file ppat.1011529.s006.docx]

**S6 Fig**

**S6 Fig. Preventing thrombin proteolysis prevents HEV replication.** Huh7 cells were electroporated with **(A)** G1 or **(B)** G3 HEV replicon RNA containing the indicated mutations at predicted thrombin cleavage junctions, in addition to the WT and GNN control replicons. Cells were harvested at the indicated times post-electroporation and luciferase activity determined. Data shown represents log_10_ of mean relative luciferase activity (n = 3 +/- SEM).
