# Supplementary material for: Thrombin cleavage of the hepatitis E virus polyprotein at multiple conserved locations is required for genome replication
Source: PLoS Pathog. 2023 Jul 21;19(7):e1011529. doi: 10.1371/journal.ppat.1011529 (PMC10395923; doi:10.1371/journal.ppat.1011529)
Supplement: S7 Fig — (A) Huh7 cells were electroporated with the WT HEV replicon RNA or GNN control replicon before the addition of AEBSF (2.5 μg / mL), antipain (50 μg / mL), chymostatin (25 μg / mL), E-64 (100 μg / mL) or leupeptin (175 μg / mL) 24 h post-electroporation. Cells were harvested at the indicated times post-electroporation and luciferase activity determined. Data shown represents log10 of mean relative luciferase activity (n = 3 +/- SEM, * = p<0.05, ** = p<0.01 compared to untreated). (B) Huh7 cells were electroporated with the WT HEV replicon RNA or GNN control replicon before the addition of apixiban (42 μM), dabigatran (42 μM), warfarin (830 μM) or DMSO solvent control (untreated) 24 h post-electroporation. Cells were harvested 96 hours post-electroporation and luciferase activity determined. Data shown represents log10 of mean relative luciferase activity at 96 h post-electroporation (n = 3 +/- SEM, * = p<0.05, ** = p<0.01 compared to untreated). (C) and (D) Huh7 cells were incubated with a serial dilution of indicated compounds for 72 hours before cell viability was measured by MTS assay. Data are expressed as mean percentage cell viability normalized to untreated controls (n = 3 +/- SEM). (DOCX) [file ppat.1011529.s007.docx]

**S7 Fig**

**S7 Fig. Pharmacological inhibition of thrombin prevents HEV replication. (A)** Huh7 cells were electroporated with the WT HEV replicon RNA or GNN control replicon before the addition of AEBSF (2.5 µg / mL), antipain (50 µg / mL), chymostatin (25 µg / mL), E-64 (100 µg / mL) or leupeptin (175 µg / mL) 24 h post-electroporation. Cells were harvested at the indicated times post-electroporation and luciferase activity determined. Data shown represents log_10_ of mean relative luciferase activity (n = 3 +/- SEM, *=p<0.05, **=p<0.01 compared to untreated). **(B)** Huh7 cells were electroporated with the WT HEV replicon RNA or GNN control replicon before the addition of apixiban (42 µM), dabigatran (42 µM), warfarin (830 µM) or DMSO solvent control (untreated) 24 h post-electroporation. Cells were harvested 96 hours post-electroporation and luciferase activity determined. Data shown represents log_10_ of mean relative luciferase activity at 96 h post-electroporation (n = 3 +/- SEM, *=p<0.05, **=p<0.01 compared to untreated). **(C)** and **(D)** Huh7 cells were incubated with a serial dilution of indicated compounds for 72 hours before cell viability was measured by MTS assay. Data are expressed as mean percentage cell viability normalized to untreated controls (n = 3 +/- SEM).
