# Supplementary material for: Thrombin cleavage of the hepatitis E virus polyprotein at multiple conserved locations is required for genome replication
Source: PLoS Pathog. 2023 Jul 21;19(7):e1011529. doi: 10.1371/journal.ppat.1011529 (PMC10395923; doi:10.1371/journal.ppat.1011529)
Supplement: S8 Fig — (A) Huh7 cells were electroporated with the G1 nLuc SGR or mock electroporated. Four days post-electroporation cells were lysed and post-nuclear supernatant used in a turbidity assay of thrombin-mediated fibrinogen to fibrin conversion (n = 3 +/- SEM). (B) Huh7 cells were electroporated with G1 HEV replicon RNA containing the indicated insertions, in addition to the WT and GNN control replicons. Cells were harvested at the indicated times post-electroporation and luciferase activity determined. Data shown represents log10 of mean relative luciferase activity (n = 2 +/- SEM). (DOCX) [file ppat.1011529.s008.docx]

**S8 Fig**

**S8 Fig. Replication of epitope tagged G1 HEV SGR. (A)** Huh7 cells were electroporated with the G1 nLuc SGR or mock electroporated. Four days post-electroporation cells were lysed and post-nuclear supernatant used in a turbidity assay of thrombin-mediated fibrinogen to fibrin conversion (n = 3 +/- SEM). **(B)** Huh7 cells were electroporated with G1 HEV replicon RNA containing the indicated insertions, in addition to the WT and GNN control replicons. Cells were harvested at the indicated times post-electroporation and luciferase activity determined. Data shown represents log_10_ of mean relative luciferase activity (n = 2 +/- SEM).
